# An AI approach for managing financial systemic risk via bank bailouts by taxpayers

**DOI:** 10.1038/s41467-022-34102-1

**Published:** 2022-11-17

**Authors:** Daniele Petrone, Neofytos Rodosthenous, Vito Latora

**Affiliations:** 1 School of Mathematical Sciences, Queen Mary University of London, E1 4NS London, UK; 2 Department of Mathematics, University College London, WC1E 6BT London, UK; 3 Dipartimento di Fisica ed Astronomia, Università di Catania and INFN, Catania, I-95123 Italy; 4 Complexity Science Hub Vienna, A-1080 Vienna, Austria

**Keywords:** Economics, Computational science, Applied mathematics

## Abstract

Bank bailouts are controversial governmental decisions, putting taxpayers’ money at risk to avoid a domino effect through the network of claims between financial institutions. Yet very few studies address quantitatively the convenience of government investments in failing banks from the taxpayers’ standpoint. We propose a dynamic financial network framework incorporating bailout decisions as a Markov Decision Process and an artificial intelligence technique that learns the optimal bailout actions to minimise the expected taxpayers’ losses. Considering the European global systemically important institutions, we find that bailout decisions become optimal only if the taxpayers’ stakes exceed some critical level, endogenously determined by all financial network’s characteristics. The convenience to intervene increases with the network’s distress, taxpayers’ stakes, bank bilateral credit exposures and crisis duration. Moreover, the government should optimally keep bailing-out banks that received previous investments, creating moral hazard for rescued banks that could increase their risk-taking, reckoning on government intervention.

## Abstract (web summary)

Systemic risk and bank bailout approaches have been the source of discussions on scientific, financial and governmental forums. An artificial intelligence technique is proposed to inform equitable bailout decisions that minimise taxpayers’ losses.

## Introduction

In times of crisis, as during the recession of 2008 or the economic disruption triggered by the COVID-19 pandemic, governments face difficult decisions regarding bailing-out strategically important companies. In particular, large banks and other financial institutions are critical for the stability of the financial system and are closely monitored by central banks and government departments. It is nowadays widely understood, that the stability of the financial system cannot be assessed focusing exclusively on each individual financial institution. The interconnections and interactions between financial institutions are at least as important in contributing to the overall dynamics (see refs. 1–5). It thus requires a broader approach to manage the risk that a considerable part of the financial system is disrupted (systemic risk). A number of regulatory boards and committees, such as the US Financial Stability Oversight Council, the European Systemic Risk Board and the Bank of England’s Financial Policy Committee, have been created in order to identify, monitor and take actions that can remove or reduce the systemic risk. They are also tasked to look for new methodologies and ideas from different disciplines to deepen their understanding of the complex phenomena involved in financial crises.

For example, in order to rescue the Royal Bank of Scotland (RBS) in 2008–2009, the UK government became the majority shareholder of the bank, purchasing shares for a total of £45.5 billion, according to ref. 6. The government achieved its objective to stabilise the financial system and no depositor in UK banks lost any money. However, the cost for taxpayers has been estimated by the Office for Budget Responsibility^7^ to be in the region of £27 billion. The price of RBS shares plummeted after the purchase and the government has since sold part of its investment at a loss. Was this governmental intervention value for money? The National Audit Office (NAO)^8^, UK’s public spending watchdog, released a report on maintaining financial stability across the UK’s banking system, analysing the governmental support to the banking sector and concluded that: If the support measures had not been put in place, the scale of the economic and social costs, if one or more major UK banks had collapsed, is difficult to envision; the support provided to the banks was therefore justified, but the final cost to the taxpayer of the support will not be known for a number of years.

A more recent example is the COVID-19 pandemic, which has had a devastating effect on economies worldwide with further forthcoming effects not fully observed yet. The quarter-over-quarter change in the US GDP fell by 31.2% in the second quarter of 2020, while the Office for National Statistics estimated that the UK economic output fell by 9.9% during the year 2020, the largest annual fall on record. The banks have so far weathered the storm, aided by improved regulations and macroprudential measures introduced in the aftermath of the global financial crisis of 2008–2009. However, no one can predict if the financial system can withstand a series of bankruptcies in the property, aviation, creative, tourism and hospitality sectors, that might ensue as the accommodating monetary policies of central banks are tapered due to the dramatic surge of inflation.

As the main concern is the systemic risk that a default entails, a network model is essential (see, e.g. refs. 9–12 for recent reviews of the financial systemic risk literature). The nodes of the network are banks or other financial institutions and their links represent mutual exposures. The connections between financial institutions can then transfer the distress amongst them (see, e.g. refs. 1, 13, 14). There is a very large literature borrowing techniques from network science (see, e.g. refs. 15, 16) and successfully applying them to the study of network resilience to external shocks in order to provide useful analyses of financial systemic risk (see, e.g. refs. 13, 17, 18). There is also a vast literature on governmental interventions in financial institutions, which spans across many different directions, such as post-bailout bank performances^19^, the bailout effects on the underwriting business^20^, on market discipline^21^ and on sovereign risk^22^, as well as the interplay amongst bailouts, banks’ risk profile and national regulation^23–26^. Our work though is related to a branch of the financial systemic risk literature that analyses interventions to limit the effects of financial crises. In particular, the relevance of bailout actions in mitigating the contagion during the financial crisis of 2008 is evidenced in ref. 27, while various network models of government interventions are proposed in refs. 28–32.

Although bank bailouts are among the most critical decisions a government can take, very few studies have addressed quantitatively the problem of assessing their convenience for the taxpayers, as we do in this paper. To be more precise, the main differences with existing literature are that: (a) our framework does not require starting the analysis with a set of banks already in default or about to default without a government intervention, hence we allow preventive actions before the network is compromised; (b) the previous literature considers the optimization of (multiple variations of) functions based on social costs, system wealth and taxpayers’ bailout money—we instead focus on minimising the loss for taxpayers during the crisis, irrespective of the size of the system’s overall wealth, which may not be directly linked to the taxpayers’ interests; (c) our modelling approach and framework fills the gaps in literature, by allowing the possibility that our dynamic network can be controlled by governments, at each and every time step, via injections of additional capital in financial institutions.

In particular, we propose a mathematical framework that allows for a quantitative comparison between different potential investments in financial institutions by the government. Our framework is based on the three following building blocks: (a) a dynamical network model of the financial system that describes the contagion mechanism between financial institutions (modelled as an increase in the probability of default of banks that have claims on failed institutions); (b) a set of allowed government interventions to control the network (investments in the capital of distressed banks); and (c) a quantitative way to assess government actions or inaction at each time step (using artificial intelligence techniques). Our main aim is to address the eventuality that a government needs to decide whether to bailout a financial institution or let it fail as its insolvency becomes more and more likely.

The contagion mechanism that we use is the impact that a bank default has on other banks. The impact can be due to: (a) direct losses from cross ownership and bilateral credit exposures (for example loans, see, e.g. refs. 33, 34), or (b) indirect losses due to fire selling of assets by defaulting banks, that would lower the market value of similar assets in the balance sheet of non-defaulting financial institutions (see, e.g. ref. 35). In all, the impact of defaults would lower the capital buffer of affected banks, thus weakening the entire network and its ability to withstand future shocks. In our model, this is accounted for by an increase of the probability of default (PD) per unit time of the banks that have claims towards the defaulted institutions. Such an existence of a PD as a characteristic of the nodes of the bilateral credit exposures network has only recently been introduced in systemic risk evaluation studies (see, e.g. refs. 36, 37).

One of our main novelties with respect to the aforementioned models is that we further allow for the network to be controlled by a government via investments in the capital of banks. Such an investment would, conversely to defaults, decrease the banks’ PD upon receiving the additional capital. The nodes’ PD thus eventually allows us to follow the evolution in time of our (controlled) dynamical model as there is a well-defined length of time during which the government can intervene to control the network. We provide the connection between the changes in each node’s PD and the changes in the amount of capital due to the impact from defaults or governmental investments via the credit risk model introduced by Merton^38^. Then, given each node’s PD, as well as the likelihood of more than one nodes defaulting simultaneously (during the same time step), which we describe by a Gaussian latent variable model, we follow the stochastic evolution of the network in time via a multi-period Monte Carlo simulation.

Even though government investments decrease the banks’ PD, they also increase the potential loss of this additional capital for the government (and taxpayers) in case of default. This creates a trade-off for the decision-makers. The main aim of the government is therefore to answer the questions of whether to invest in financial institutions at each time step, which financial institution(s) to invest in and how much to invest, in order to achieve the minimum expected taxpayers’ loss during a crisis.

To that end, we model the system’s evolution as a Markov Decision Process (MDP) (see ref. 39), where the actions (controls) are government investments in the capital of the banks at each time step, and the dynamics and negative rewards (losses) are linked to the financial network dynamics, each node’s (controlled) probability of default, total asset and previous government investments (the sequence of governmental stochastic controls). However, our MDP is both challenging to define in this setup and (even more) challenging to solve, given its following main characteristics: (a) the MDP state definition is remarkably complex since it depends on all the parameters of the network at each specific time; (b) the low probability of default of each node in the network translates in a high probability of not receiving any reward signals to learn the best action, (c) the enormous number of successor states (even in very simple networks) would normally make standard computations impossible and standard methods non-feasible.

In order to overcome these challenges, we develop an artificial intelligence technique that uses a variation of the Fitted Value Iteration algorithm (see, e.g. refs. 40, 41) with bespoke characteristics that are uniquely constructed to solve our MDP. To be more precise, we: (a) devise a particular value function parametrisation, representing the sum of the expected direct losses per node and remaining time steps; (b) implement a learning process backwards in time from the end of the stochastic episode (financial crisis) where the value function is known to be zero; and (c) use an ingenious duality between the dynamics of the financial network (our nodes’ default modelling) and the MDP rewards and transition probabilities, to reduce drastically the number of terms in many critical expressions—in particular, we devise a technique for rewriting these expressions in terms of (the remarkably smaller) number of non-defaulted nodes rather than successor states (according to standard theory), hence allowing their computation. Our methodology allows us to assess the optimality of government decisions—no investment versus different types and amounts of investment—and conclude the optimal government actions per time step and state of the network.

The introduced framework has a high potential for becoming an important tool for central banks and governments, whose budget, data and resources could allow for a professional calibration of our model. The mathematical assessment and dynamic optimisation of bank bailout decisions from a taxpayer’s standpoint could be a valuable quantitative tool in their diverse toolbox (make new bailout decisions, apply on selected bailout cases to evaluate results, learn from past experiences and actual happenings). Furthermore, our proposed methodology could have not only a practical impact to assess government interventions, but also a significant impact on the scientific community aiming at tackling problems of stochastic control in dynamic networks with few reward signals, as the one we solve in this paper.

## Results

One of the main results of our paper is the introduction of a mathematical framework, that allows governments to assess the convenience to intervene with bailout investments in distressed banks’ equity and optimise their decisions. In the following subsections, we first present the network model of financial institutions, its dynamics and contagion mechanism, and then propose an MDP based on the network, which will be used to model government interventions on bailed-out financial institutions. After formulating the problem, we proceed with a methodology for solving the MDP and some of the mathematical technicalities. Finally, we implement our framework and methodology on two case studies and present our findings.

### Network of financial institutions

We consider a network whose set ${{{{{{{\mathcal{I}}}}}}}}=\{1,\ldots,N\}$ of nodes represents financial institutions. Each node $i\in {{{{{{{\mathcal{I}}}}}}}}$ is characterised at time *t* by a probability of default *PD*_*i*_(*t*)∈(0,1) per time interval Δ*t*, a total asset *W*_*i*_(*t*) and an equity *E*_*i*_(*t*), that is the capital used by node *i* as a buffer to withstand financial losses, satisfying *E*_*i*_(*t*) ≤ *W*_*i*_(*t*).

The edge (*i*, *j*) of the network represents the exposure of node *i* to the default of node *j* where $i\,\ne\, j\in {{{{{{{\mathcal{I}}}}}}}}$. Each edge (*i*, *j*) is associated to a numerical value *w*_*ij*_ which depends on the contagion channels considered. For example, we can consider only credit exposures or also the impact due to fire sales of common assets. Regarding credit exposures, most of the times only aggregated values are available, e.g. the total amount of inter-banking assets and liabilities for each node, and in such cases, bespoke algorithms are used to infer the network of bilateral exposures (see, e.g. refs. 37, 42). To take into account government interventions aimed at limiting the overall losses, we use an adaptation of the PD model introduced in ref. 37 by extending it to allow the possibility for the nodes to incur also positive shocks, via investments in the nodes, rather than just negative shocks due to the default of other nodes. The focus of this paper is also radically different from the one in ref. 37, which focuses on the losses sustained by private investors, since we are here exclusively interested in the losses incurred by the taxpayers. In the following, we will measure the time in discrete time steps that are multiples of Δ*t*, i.e. *t* + 1 is equivalent to *t* + Δ*t*.

We define the total impact *I*_*i*_(*t*) on node *i* at time *t*, due to the default of other nodes $j\in {{{{{{{\mathcal{I}}}}}}}}\setminus \{i\}$ in the network and their exposure *w*_*ij*_, by

1 $${I}_{i}(t): \!\!=\mathop{\sum}\limits_{j\in {{{{{{{\mathcal{I}}}}}}}}\setminus \{i\}}{w}_{ij}{\delta }_{j}(t),\quad \,{{\mbox{for all}}}\,i\in {{{{{{{\mathcal{I}}}}}}}},$$

where

$${\delta }_{j}(t)=\left\{\begin{array}{l}1,\quad \,{{\mbox{if node}}}\,\,j\,{{\mbox{defaults at time}}}\,\,t,\\ 0,\quad {{{{{{{\rm{otherwise}}}}}}}}.\hfill \end{array}\right.$$

The mechanism by which defaults will occur at each time step *t*, yielding *δ*_⋅_(*t*) = 1, will be constructed towards the end of this section using all network information, including the probabilistic framework up to time *t*. The impact *I*_*i*_(*t*) represents a loss for the total asset *W*_*i*_, which in turn decreases also the equity *E*_*i*_ of node *i*, hence reducing their value at time *t* + 1. This can be seen from the accounting equation for each node *i*, namely

2 $${W}_{i}(t)={E}_{i}(t)+{B}_{i}(t),$$

which states that the total asset *W*_*i*_ is always equal, at all times, to the equity *E*_*i*_ plus the total liability *B*_*i*_. Note that *B*_*i*_ is not affected by the losses as it is comprised of loans from other banks, deposits, etc., that are due in full unless the bank *i* defaults. Hence, we have

3 $$\Delta {W}_{i}(t)=\Delta {E}_{i}(t),$$

where we define Δ*X*_*i*_(*t*) ≔ *X*_*i*_(*t* + 1)−*X*_*i*_(*t*). Taking into account also the potential increase Δ*J*_*i*_(*t*) in the cumulative investment *J*_*i*_(*t*) of the government in node *i* up to time *t*, which will in turn increase the values of the total asset *W*_*i*_ and equity *E*_*i*_ of node *i* at time *t* + 1, we can write

4 $${W}_{i}(t+1)={W}_{i}(t)-{I}_{i}(t)+\Delta {J}_{i}(t)\qquad {{{{{{{\rm{and}}}}}}}}\qquad {E}_{i}(t+1)={E}_{i}(t)-{I}_{i}(t)+\Delta {J}_{i}(t).$$

The probability of default *PD*_*i*_(*t*) of node *i* is increased by the impact *I*_*i*_(*t*) at time *t*, since part of the capital buffer (equity *E*_*i*_) is lost, and decreased by the potential investment Δ*J*_*i*_(*t*), which in turn grows the capital buffer. In order to model the effect of the impact *I*_*i*_(*t*) and potential investment Δ*J*_*i*_(*t*) on *PD*_*i*_(*t*), we use here the credit risk model introduced by Merton^38^. Alternatively, it is possible to use the first passage model introduced by Black and Cox^43^. The implied probability of default *PDM* is therefore calculated as a function of the parameters of each node:

5 $$PDM(W,E,\mu,\sigma ): \!\!=1-\Phi \left(\left(\log \frac{W}{W-E}+\mu -\frac{{\sigma }^{2}}{2}\right)\bigg/\sigma \right),$$

where the term *W*−*E* represents the total liability *B* of each bank, Φ is the univariate standard Gaussian distribution, *μ* is the drift (expected growth rate) and *σ* is the volatility of the geometric Brownian motion associated to the total asset *W* in the Merton model. We then use (5) to obtain the probability of default of node *i*,

6 $$P{D}_{i}(t):\!=\max \left\{PDM({W}_{i}(t),{E}_{i}(t),{\mu }_{i},{\sigma }_{i}),PD{M}_{i}^{\,\,floor}\right\},$$

where we introduce the fixed number $PD{M}_{i}^{floor}$, whose purpose is to exclude unreasonably low probabilities of default, essentially acting as a lower bound of the *PD*_*i*_. A lower bound $PD{M}_{i}^{floor}$ is necessary, as no matter how well a bank *i* is capitalised against losses, it can still default due to extreme events such as natural disasters, political revolutions, sovereign defaults, etc. Without $PD{M}_{i}^{floor}$, the government would underestimate the actual probability of default and would tend to invest more capital than it is convenient. As an example of calibration of this parameter, we can follow the standard assumption that the *PD*_*i*_ of a bank $i\in {{{{{{{\mathcal{I}}}}}}}}$ is greater or equal to the probability of default of the country where it is based in. In this context, the $PD{M}_{i}^{floor}$ would be the probability of the country hosting bank *i* to default on its debt.

Now, if node *i* loses an amount of capital *I*_*i*_(*t*) greater than its capital buffer (equity *E*_*i*_(*t*)), at some time *t*, the total asset *W*_*i*_(*t*) becomes less than its liability *B*_*i*_(*t*) and it is convenient for the shareholders to exercise their option to default. In practice, when this occurs, we set *PD*_*i*_(*t* + 1) = 1 and node *i* will default at time *t* + 1. Moreover, recall that node *i* may also default at any time *t* with probability *PD*_*i*_(*t*) due to its own individual characteristics given by (6); see also the default mechanism described at the end of this subsection

Now, when node *i* defaults, we denote by *LGD*_*i*_ the loss given default of node *i*, which is a fixed number representing the percentage of the cumulative investments *J*_*i*_ on node *i* by the government, that cannot be recovered after a default. In case of default of node *i*, we further assume that in addition to the aforementioned loss of investments, the taxpayers’ loss *L*_*i*_ is also comprised of a fixed percentage *α*_*i*_ (for convenience) of the total asset *W*_*i*_ of the node *i*. That is, the taxpayers’ overall loss *L*_*i*_(*t*) at time *t* is given by

7 $${L}_{i}(t):\!\!\!={\alpha }_{i}{W}_{i}(t)+LG{D}_{i}\,{J}_{i}(t).$$

To complete our framework, we need to specify the probability of more than one default happening during the same time step, given the *PD*_*i*_ of each node *i* obtained as in (6). For example, if the nodes were independent, the probability of nodes *i* and *j* defaulting at the same time step, denoted by *PD*_[*ij*]_, would be the product of the individual probabilities *PD*_*i*_ and *PD*_*j*_. In this paper, we allow nodes to depend on each other and use a Gaussian latent variable model (see, e.g. ref. 44) to calculate the probabilities of simultaneous defaults of two or more nodes. To be more precise, the probability of a finite subset of nodes $\{i,j,k,\ldots \}\subseteq {{{{{{{\mathcal{I}}}}}}}}$ of the network defaulting at the same time, is given by

8 $$P{D}_{[i,\, j,k,\ldots ]}: \!\!={\int}_{D}{\Phi }_{N}^{\prime}({{{{{{{\bf{u}}}}}}}};\Sigma )d{{{{{{{\bf{u}}}}}}}},$$

where ${\Phi }_{N}^{\prime}$ is the standardised multivariate Gaussian density function, with zero mean and a symmetric correlation matrix Σ ∈ [−1, 1]^*N*×*N*^, given by

9 $${\Phi }_{N}^{\prime}({{{{{{{\bf{u}}}}}}}};\Sigma ): \!\!=\frac{\exp \{-\frac{1}{2}{{{{{{{{\bf{u}}}}}}}}}^{T}{\Sigma }^{-1}{{{{{{{\bf{u}}}}}}}}\}}{\sqrt{{(2\pi )}^{n}\,|\Sigma|}}$$

and ∣Σ∣ is the determinant of Σ. We further note that the integration domain *D* in (8) is the Cartesian product of the intervals $[-\infty,{\Phi }_{1}^{-1}(P{D}_{l})]$ for each node *l* that belongs to the set of defaulting nodes {*i*, *j*, *k*,…}, and the intervals [−*∞*,*∞*] for the remaining non-defaulting nodes, where ${\Phi_{1}}$ is the univariate standard Gaussian distribution.

We are now ready to present the mechanism according to which nodes can default based on their individual characteristics. To be more precise, at each time step *t*, we first sample values (*x*_1_,…,*x*_*N*_) of the random vector $X={({X}_{1},{X}_{2},\ldots,{X}_{N})}$ with the multivariate Gaussian distribution of the underlying Gaussian latent variable model mentioned above. Then, we assume that node *i* defaults according to the rule:

10 $${x}_{i}\, < \,{\Phi }_{1}^{-1}(P{D}_{i}(t))\quad \ \iff \ \quad {\delta }_{i}(t)=1.$$

### The banks bailout problem as a Markov Decision Process

In this subsection, we describe the government decisions of bailing out banks as a Markov Decision Process (MDP) driven by our framework described in the previous subsection. We firstly assume that the government estimated that the crisis will likely be over at time *M*, where each time step could be interpreted to reflect the contagion effect, which occurs across periods in our model, or the governmental review frequency of the possibility to invest in financial institutions in the midst of a crisis. In any case, recalling that the government invests in the equity of banks and other financial institutions, we assume that it will be able to sell the acquired shares to the private sector, after the end of the crisis, for a price that is similar to the purchasing one. In reality, this price is directly linked to the expectation of the future dividends to be paid by the surviving bank. The government could then realise a profit on these investments, after a considerable rise in the aggregate stock market at the end of the crisis, from time *M* + 1 onwards (see ref. 45 for a relevant research investigation), or even make a loss. Clearly, any scenario would affect the effective taxpayers loss. In this paper though, we focus solely on the minimisation of taxpayers losses due to bailouts and bank defaults during the crisis episode (time 0 to *M*), by assuming a neutral realised return on investments in surviving institutions beyond time *M*.

We define the 4-tuple (*S*, *A*_*s*_, *P*_*a*_, *R*_*a*_) of the set *S* of all the states of the dynamic network (namely the state space in which the processes’ evolution takes place, leading to all possible configurations of the financial system), set *A*_*s*_ of all actions available to the government from state *s* ∈ *S*, transition probabilities ${P}_{a}(s,{s}^{\prime})=P({s}_{t+1}={s}^{\prime}\,|\,{s}_{t}=s,{a}_{t}=a)$ between state s at any time *t* and state ${s}^{\prime}$ at time *t* + 1 having taken action *a* ∈ *A*_*s*_ at time *t*, and rewards ${R}_{a}(s,{s}^{\prime})$ (negative losses in our model) received after taking action *a* at any time *t* while being at state *s* and landing in state ${s}^{\prime}$ at time *t* + 1, where $s,{s}^{\prime}\in S$. Furthermore, we consider a constant discount factor *γ* with 0 ≤ *γ* < 1, so that rewards obtained sooner are more relevant. The discounted cumulative reward *G* from time step *t* until the end of crisis (recall that a full episode consists of *M* time steps) is therefore defined by

11 $$G(t): \!\!\!=\mathop{\sum }\limits_{u=t}^{M-1}{\gamma }^{u-t}{R}_{{a}_{u}}({s}_{u},{s}_{u+1}^{\prime}).$$

In the remaining of this subsection, we expand on the 4-tuple (*S*, *A*_*s*_,*P*_*a*_,*R*_*a*_) that defines our MDP and formulate our stochastic control problem.

Firstly, we introduce the MDP states. The states *s*_*t*_ ∈ *S*, at each time *t*, in which the financial system may end up, are defined by three main pillars: (a) all the parameters of the network (*W*_*i*_(*t*), *E*_*i*_(*t*), *PD*_*i*_(*t*), *J*_*i*_(*t*), *LGD*_*i*_, *α*_*i*_, *μ*_*i*_, *σ*_*i*_, *w*_*ij*_, Σ_*ij*_, for *i*, *j* ∈ {1,…,*N*}, where *w*_*ii*_ = 0), (b) an indexed set ${{{{{{{{\mathcal{I}}}}}}}}}_{def}(t)\subseteq {{{{{{{\mathcal{I}}}}}}}}$ containing all defaulted nodes prior to time *t* and (c) the time to maturity *M*−*t*.

Secondly, we introduce the MDP actions and governmental policies. The MDP actions ${a}_{t}\in {A}_{{s}_{t}}$ in our model are the control variables of the government when trying to minimise the losses of the network (i.e. maximise the expected *G* in (11)). They correspond to injections of capital ${a}_{t}\to {{{{{{{\boldsymbol{\Delta }}}}}}}}{{{{{{{{\bf{J}}}}}}}}}^{{{{{{{{\bf{a}}}}}}}}}(t): \!\!=(\Delta {J}_{1}^{a}(t),\Delta {J}_{2}^{a}(t),\ldots,\Delta {J}_{N}^{a}(t))$ increasing the government’s investments in the nodes (1, 2,…,*N*), affecting their total wealth and equity according to (4), whose updated (increased) values are denoted by

12 $${J}_{i}^{a}(t): \!\!={J}_{i}(t)+\Delta {J}_{i}^{a}(t),\quad {W}_{i}^{a}(t): \!\!={W}_{i}(t)+\Delta {J}_{i}^{a}(t)\quad {{{{{{{\rm{and}}}}}}}}\quad {E}_{i}^{a}(t): \!\!={E}_{i}(t)+\Delta {J}_{i}^{a}(t).$$

These additional resources on one hand, make the nodes more resilient, hence diminishing their updated probability of default $P{D}_{i}^{a}$ via (5), (6), namely

13 $$P{D}_{i}^{a}(t): \!\!\!=\max \{PDM({W}_{i}^{{a}}(t),\,{E}_{i}^{a}(t),\,{\mu }_{i},\,{\sigma }_{i}),\,PD{M}_{i}^{floor}\},$$

leading to (statistically) less defaults due to the updated default mechanism (recall (10)) given by

14 $${x}_{i}\, < \,{\Phi }_{1}^{-1}\left(P{D}_{i}^{a}(t)\right)\quad \ \iff \ \quad {\delta }_{i}^{a}(t)=1,$$

and consequently to an updated (statistically decreased) total impact

15 $${I}_{i}^{a}(t): \!\!=\mathop{\sum}\limits_{j\in {{{{{{{\mathcal{I}}}}}}}}\setminus \{i\}}{w}_{ij}{\delta }_{j}^{a}(t),\quad \,{{\mbox{for all}}}\,\,i\in {{{{{{{\mathcal{I}}}}}}}}.$$

On the other hand, these resources will be at risk in case of node *i* defaulting at time *t*, since the aforementioned (increased) cumulative investment ${J}_{i}^{a}$ and total wealth ${W}_{i}^{a}$ will both contribute towards an increased updated taxpayers’ overall loss ${L}_{i}^{a}$ given via (7) by

16 $${L}_{i}^{a}(t): \!\!={\alpha }_{i}{W}_{i}^{a}(t)+LG{D}_{i}\,{J}_{i}^{a}(t).$$

Recalling that each action ${a}_{t}\in {A}_{{s}_{t}}$ depends on the current state *s*_*t*_ at any time *t*, we denote the government policy by a function *π*(*s*_*t*_) → *a*_*t*_ that indicates which action to take at each state. A policy that minimises the expected network losses is called optimal policy and is denoted by π_*_, while the action ${a}_{t}^{*}$ returned by π_*_ given a state *s*_*t*_ (i.e. ${\pi }_{*}({s}_{t})\to {a}_{t}^{*}$) is then called the optimal action for that state.

Note that, the model can easily incorporate also the nature of governmental equity injections (asking banks to repay debt, or invest in safer assets to hedge against future losses, or change their strategy in exchange for funding), that would eventually lead to updated ${\mu }_{i}^{a}(t)={\mu }_{i}(\Delta {J}_{i}^{a}(t))$ and ${\sigma }_{i}^{a}(t)={\sigma }_{i}(\Delta {J}_{i}^{a}(t))$ affecting only the resulting updated probability of default $P{D}_{i}^{a}(t)$ in (13), while the rest of our framework and methodology would remain intact.

Thirdly, we introduce the MDP transition probabilities. Within our framework, a node that has defaulted does not contribute to future losses and cannot become active again, i.e. the cardinality of the set of defaulted nodes $|{{{{{{{{\mathcal{I}}}}}}}}}_{def}(t)|$ is a non-decreasing function of time *t*. Hence the transition probability ${P}_{a}(s,{s}^{\prime})$ from state ${s}$ to ${s}^{\prime}$ will be non-zero only for states ${s}^{\prime}$ that: (a) have the same number or more defaulted nodes than state *s*; (b) are “reachable”, in the sense that their characteristics *PD*_*i*_(*t* + 1), *W*_*i*_(*t* + 1) and *E*_*i*_(*t* + 1), for $i\in {{{{{{{\mathcal{I}}}}}}}}\setminus {{{{{{{{\mathcal{I}}}}}}}}}_{def}(t+1)$ (the remaining active nodes in ${s}^{\prime}$) take values that are coherent with eqs. (4)–(6) after calculating the impacts *I*_*i*_(*t*) from the newly defaulted nodes $i\in {{{{{{{{\mathcal{I}}}}}}}}}_{def}(t+1)\setminus {{{{{{{{\mathcal{I}}}}}}}}}_{def}(t)$ at time *t*. For an example on how to identify these so-called reachable states, we refer to the Reachable MDP states example in the Methods section.

Then, for all states ${s}_{t+1}^{\prime}$ with a non-zero transition probability ${P}_{{a}_{t}}({s}_{t},{s}_{t+1}^{\prime})$, we can calculate the latter via the Gaussian latent variable model (see also (8), (9)). To be more precise, given the government investments relative to action *a*_*t*_ at state *s*_*t*_ and time *t*, we use the updated ${J}_{i}^{a}(t),{W}_{i}^{a}(t),{E}_{i}^{a}(t)$ and $P{D}_{i}^{a}(t)$ from (12)–(13) to calculate the transition probability via (see also (8)) the following integral:

17 $${P}_{{a}_{t}}({s}_{t},{s}_{t+1}^{\prime}): \!\!={\int}_{D}{\Phi }_{|{{{{{{{\mathcal{I}}}}}}}}\setminus {{{{{{{{\mathcal{I}}}}}}}}}_{def}(t)|}^{\prime}({{{{{{{\bf{u}}}}}}}};{\Sigma }_{sub}(t))d{{{{{{{\bf{u}}}}}}}},$$

where ${\Phi }^{\prime}$ is the density given by (9) with dimension equal to the cardinality of the set of surviving nodes $|{{{{{{{\mathcal{I}}}}}}}}\setminus {{{{{{{{\mathcal{I}}}}}}}}}_{def}(t)|\le N$. Upon recalling the updated version of the default mechanism in (14), the integration domain *D* in (17) is given by the Cartesian product of the intervals $[-\infty,{\Phi }_{1}^{-1}(P{D}_{i}^{a})]$ for the additional defaulted nodes $i\in {{{{{{{{\mathcal{I}}}}}}}}}_{def}(t+1)\setminus {{{{{{{{\mathcal{I}}}}}}}}}_{def}(t)$ and the intervals $[{\Phi }_{1}^{-1}(P{D}_{i}^{a}),\infty ]$ for all the remaining active nodes $i\in {{{{{{{\mathcal{I}}}}}}}}\setminus {{{{{{{{\mathcal{I}}}}}}}}}_{def}(t+1)$ at state ${s}_{t+1}^{\prime}$. The Σ_*sub*_(t) is the sub-matrix of the original correlation matrix Σ after removing the rows and the columns corresponding to defaulted nodes $i\in {{{{{{{{\mathcal{I}}}}}}}}}_{def}(t)$ at state *s*_*t*_.

Thus, we observe that in our model, the transition probabilities depend exclusively on the government investments *a*_*t*_, the resulting financial institutions’ probability of default $P{D}_{i}^{a}$ and the correlation structure Σ_*ij*_ with $i,\,j\in {{{{{{{\mathcal{I}}}}}}}}\setminus {{{{{{{{\mathcal{I}}}}}}}}}_{def}(t)$ which links the financial institutions in the network.

Fourthly, we introduce the MDP rewards. In our model the rewards take non-positive values, since their overall maximisation has to translate for our MDP into the minimisation of the potential overall taxpayers’ losses ${L}_{i}^{a}(t)$ in (16) for all nodes $i\in {{{{{{{\mathcal{I}}}}}}}}\setminus {{{{{{{{\mathcal{I}}}}}}}}}_{def}(t)$ after taking action *a*_*t*_ at each time *t*. Namely, in light of the updated default mechanism (14), we define the reward at time *t* by

18 $${R}_{{a}_{t}}({s}_{t},{s}_{t+1}^{\prime}): \!\!=-\mathop{\sum}\limits_{i\in {{{{{{{\mathcal{I}}}}}}}}\setminus {{{{{{{{\mathcal{I}}}}}}}}}_{def}(t)}\left({\alpha }_{i}{W}_{i}^{a}(t)+LG{D}_{i}\,{J}_{i}^{a}(t)\right){\delta }_{i}^{a}(t),$$

where only the nodes defaulting at time *t* after taking action *a*_*t*_, i.e. having ${\delta }_{i}^{a}(t)=1$, contribute to the sum of losses. This means that the reward at time *t* can be 0, in case there are no additional defaults occurring at time *t*.

Finally, we are ready to define the optimal value function and present the stochastic control problem formulation. By doing so, we will formalise the main aim of the government, which is the minimisation of taxpayers’ losses during the crisis episode. We therefore need our model to indicate if the government should intervene and if so, which amount it should invest for a given configuration of the financial system to achieve its aforementioned goal. This mathematically translates to the government aiming at finding the optimal actions ${a}_{t}^{*}\in {A}_{{s}_{t}}$, or equivalently the optimal policy π_*_, for successive time steps, starting from any time *t* and any possible state *s*_*t*_ of the dynamic network until the end of the episode at time *M*, in order to maximise the expected discounted cumulative reward *G*(*t*) given by (11).

The optimal value function V_*_(*s*_*t*_) is then defined as the expected discounted cumulative reward *G*(*t*) starting from state *s*_*t*_ at time *t* and following the aforementioned optimal policy π_*_, given in light of the definition of rewards (in particular their expression in (18)) by

19 $${V}_{*}({s}_{t}): \! 	={E}_{{\pi }_{*}}[G(t)|{s}_{t}]={E}_{{\pi }_{*}}\left[\mathop{\sum }\limits_{u=t}^{M-1}{\gamma }^{u-t}{R}_{{a}_{u}}({s}_{u},{s}_{u+1}^{\prime}) \bigg| {s}_{t}\right]\\ 	=-{E}_{{\pi }_{*}}\left[\mathop{\sum }\limits_{u=t}^{M-1}{\gamma }^{u-t}\mathop{\sum}\limits_{i\in {{{{{{{\mathcal{I}}}}}}}}\setminus {{{{{{{{\mathcal{I}}}}}}}}}_{def}(u)}\left({\alpha }_{i}{W}_{i}^{a}(u)+LG{D}_{i}\,{J}_{i}^{a}(u)\right){\delta }_{i}^{a}(u)\bigg| \,{s}_{t}\right] \\ 	 \forall \quad t\,\in [0,M-1]\quad {{{{{{{\rm{and}}}}}}}}\quad {V}_{*}({s}_{M}): \!\!=0,$$

where the latter definition follows due to the time step *M* signifying the end of the crisis episode, when the government can sell all its shares in the banks, thus incurring no additional losses.

Given the definition of π_*_, the optimal value function V_*_(*s*_*t*_) represents the maximum expected discounted cumulative reward, which translates into the minimum expected discounted taxpayers’ loss, that can be obtained amongst all possible policies *π* starting from *s*_*t*_,

20 $${V}_{*}({s}_{t}) 	=\mathop{\max }\limits_{\pi }{E}_{\pi }[G(t)|{s}_{t}] \\ 	=-\mathop{\min }\limits_{\pi }{E}_{\pi }\left[\left.\mathop{\sum }\limits_{u=t}^{M-1}{\gamma }^{u-t}\mathop{\sum}\limits_{i\in {{{{{{{\mathcal{I}}}}}}}}\setminus {{{{{{{{\mathcal{I}}}}}}}}}_{def}(u)}\left({\alpha }_{i}{W}_{i}^{a}(u)+LG{D}_{i}{J}_{i}^{a}(u)\right){\delta }_{i}^{a}(u)\right|{s}_{t}\right].$$

The optimal action value function Q_*_(*s*_*t*_, *a*_*t*_) is the expected discounted cumulative reward we obtain, if we first take action *a*_*t*_ while being at state *s*_*t*_ and then follow the optimal policy π_*_ for any of the successive steps from *t* + 1 until the end of the episode *M*. Mathematically, this is defined by

21 $${Q}_{*}({s}_{t},{a}_{t}): 	={E}_{{\pi }_{*}}[G(t)|{s}_{t},{a}_{t}] \\ 	=E\left[\left.{R}_{{a}_{t}}({s}_{t},{s}_{t+1}^{\prime})\right|{s}_{t},{a}_{t}\right]+{E}_{{\pi }_{*}}\left[\left.\mathop{\sum }\limits_{u=t+1}^{M-1}{\gamma }^{u-t}{R}_{{a}_{u}}({s}_{u}^{\prime},{s}_{u+1}^{\prime})\right|{s}_{t},{a}_{t}\right].$$

Similarly to the optimal value function, Q_*_(*s*_*t*_, *a*_*t*_) represents the maximum expected cumulative reward that can be obtained when starting from *s*_*t*_ and after taking action *a*_*t*_ at time *t*.

The contribution of the optimal action value function in providing the desired quantitative evaluation required for implementing the model in real-life scenarios is twofold. Firstly, notice that finding Q_*_ is equivalent to solving the MDP, since the optimal action ${a}_{t}^{*}$ for each state *s*_*t*_ (hence the optimal policy π_*_) can be obtained by

22 $${a}_{t}^{*}=\mathop{{{{{{{{\rm{argmax}}}}}}}}}\limits_{{a}_{t}}\,{Q}_{*}({s}_{t},{a}_{t}).$$

Secondly, we use Q_*_ in order to quantify the convenience to intervene Conv(*s*_*t*_) for the government at each state *s*_*t*_ and any time *t*, in the forthcoming model implementations. To be more precise, we define by Conv(*s*_*t*_) the difference between the optimal action value function corresponding to the best governmental intervention and the optimal action value function associated to ${a}_{t}^{0}$, which denotes the inaction (no investments) at time *t*, when being at the state *s*_*t*_, i.e.

23 $${{{{{{{\rm{Conv}}}}}}}}({s}_{t}): \!\!=\mathop{\max }\limits_{{a}_{t}\in {A}_{{s}_{t}}\setminus \{{a}_{t}^{0}\}}\{{Q}_{*}({s}_{t},{a}_{t})\}-{Q}_{*}({s}_{t},{a}_{t}^{0}).$$

### AI technique to solve the MDP

In this subsection, we present our artificial intelligence technique to solve the MDP, driven by our dynamic network of the financial system that can be controlled by a regulator in view of minimising the expected taxpayers’ loss.

We firstly recall a standard relationship between optimal value functions and action value functions in MDPs. Observe that the two terms on the right-hand side in the definition (21) of the optimal action value function Q_*_(*s*_*t*_, *a*_*t*_) are first the immediate expected reward at time *t* due to taking action *a*_*t*_ and second the optimal expected discounted cumulative reward from time *t* + 1 onwards. We can therefore rewrite Q_*_(*s*_*t*_,*a*_*t*_) from (21) in terms of the transition probabilities (recall (17)) and the future optimal value functions ${V}_{*}({s}_{t+1}^{\prime})$ defined in (19), in the form

24 $${Q}_{*}({s}_{t},{a}_{t})=\mathop{\sum}\limits_{{s}_{t+1}^{\prime}}{P}_{{a}_{t}}({s}_{t},{s}_{t+1}^{\prime})({R}_{{a}_{t}}({s}_{t},{s}_{t+1}^{\prime})+\gamma {V}_{*}({s}_{t+1}^{\prime})).$$

It is also straightforward to see from the definitions (19) and (21) of the optimal value function V_*_(*s*_*t*_) and action value function Q_*_(*s*_*t*_,*a*_*t*_), respectively, that

25 $${V}_{*}({s}_{t})=\mathop{\max }\limits_{{a}_{t}}\,{Q}_{*}({s}_{t},{a}_{t}),$$

i.e. the maximum expected discounted cumulative reward from *s*_*t*_ is the one corresponding to the maximum value of Q_*_ amongst all available potential actions ${a}_{t}\in {A}_{{s}_{t}}$ at time *t*. Substituting the expression of (24) in (25) thus gives the Bellman optimality equation

26 $${V}_{*}({s}_{t})=\mathop{\max }\limits_{{a}_{t}\in {A}_{{s}_{t}}}\left\{\mathop{\sum}\limits_{{s}_{t+1}^{\prime}}{P}_{{a}_{t}}\left({s}_{t},{s}_{t+1}^{\prime}\right)\left({R}_{{a}_{t}}\left({s}_{t},{s}_{t+1}^{\prime}\right)+\gamma {V}_{*}\left({s}_{t+1}^{\prime}\right)\right)\right\}.$$

Given that we have a complete description of our MDP (in particular, we have the transition probabilities ${P}_{{a}_{t}}({s}_{t},{s}_{t+1}^{\prime})$ and the rewards ${R}_{{a}_{t}}({s}_{t},{s}_{t+1}^{\prime})$), we could in theory enumerate all possible states, use Dynamic Programming and the Value Iteration algorithm (see ref. 46) to solve our stochastic control problem. This would essentially involve finding V_*_ using the Bellman optimality equation in (26) and then calculating Q_*_ via (24), thus solving the MDP. However, applying this standard theory is not a scalable/feasible approach due to (a) the complexity of the MDP states and (b) the enormous number of successor states ${s}^{\prime}$ (for all but trivial networks), making standard computations impossible.

We, therefore, propose in this paper an approach to solve the MDP, which involves the use of a variation of the Fitted Value Iteration algorithm (see, e.g. refs. 40, 41) with bespoke characteristics uniquely constructed in our artificial intelligence technique.

Our method consists of the following four steps:

(i) Devise a parametric representation ${\overline{V}}_{*}(s,\beta )$ for the optimal value function V_*_(*s*), where *β* is a placeholder for a set of parameters to fit (see our construction in the Value function approximation subsection, Methods section);
(ii) Use ${\overline{V}}_{*}(s,\beta )$ to devise a parametric representation ${\overline{Q}}_{*}(s,a,\beta )$ for the optimal action value function Q_*_(*s*, *a*) in (24) (see the Action value function approximation subsection, Methods section, for its derivation and our technique to calculate it);
(iii) Use ${\overline{Q}}_{*}(s,a,\beta )$ for the right-hand side of (25) to obtain an approximate Bellman optimality equation in order to fit *β* via a learning process (see our technique in the Learning process subsection, Methods section), which will eventually give ${V}_{*}(s)\,\approx \,{\overline{V}}_{*}(s,{\beta }^{\;fit})$;
(iv) Finally, use ${\overline{V}}_{*}(s,{\beta }^{\;fit})$ to calculate ${\overline{Q}}_{*}(s,a,{\beta }^{\;fit})\,\approx \,{Q}_{*}(s,a)$, and hence solve the MDP as in the Optimal solution of the MDP subsection, Methods section.

Each one of the aforementioned steps bares its own difficulties and technical obstacles, which we overcome in the analysis presented in the aforementioned subsections of the Methods section.

In the following subsections, we use our artificial intelligence technique to solve the MDP in two implementation case studies. We show how our model works and obtain qualitative results on the optimal bailout decision problem faced by governments. A professional calibration of our model would require the effort and firepower of a central bank or a government office, and access to sensitive data. Nonetheless, by exploring these two case studies, we provide useful insights for whether and when taxpayers should fund bank bailouts.

### Setup of implementation case studies

To illustrate how our method works and its potential, we apply our model on both a synthetic homogeneous network (Krackhardt kite graph) and a real network of the European global systemically important institutions. Before we present our case studies, we assign values to a set of parameters, that are common to both case studies (unless otherwise specified). We consider a crisis episode that will last for *M* = 7 time steps, a discount factor *γ* = 0.98 and an initial government investment *J*_*i*_(0) = 0 for each node (bank or other financial institution) $i\in {{{{{{{\mathcal{I}}}}}}}}$. Moreover, we assume the percentages *α*_*i*_ of wealth loss upon default of node *i* to be all the same, i.e. *α*_*i*_ = *α*, and we conservatively assume that the expected value of the total wealth’s return is *μ*_*i*_ = 0, for all $i\in {{{{{{{\mathcal{I}}}}}}}}$. Recall that, we are considering equity investments by the government that can be recovered, in case of default, only after all the depositors and bond holders are satisfied. Hence, we assume that the government loses all its investments in case of default, i.e. *LGD*_*i*_ = 1, for all $i\in {{{{{{{\mathcal{I}}}}}}}}$. If the government is allowed to use other means besides equity investments, e.g. bond investments, then *LGD*_*i*_ ∈ (0, 1) (see ref. 47 for a study on senior and subordinated recovery rates). However, this would imply a softer effect on the solvency issues and require a modification of the probability of default formula in (13), since the investment would not be directly affecting the equity (12) anymore. In order to take into account the average correlation between financial institutions, we use a homogeneous correlation matrix for our nodes, which is set to Σ_*ij*_ = 0.5 for $i\ne j\,\in \,{{{{{{{\mathcal{I}}}}}}}}\setminus {{{{{{{{\mathcal{I}}}}}}}}}_{def}$ following ref. 48. The volatility *σ*_*i*_ of the total wealth’s return for each $i\in {{{{{{{\mathcal{I}}}}}}}}$, is calculated at time 0 by inverting (5) using the initial (known) values of *PD*_*i*_(0), *E*_*i*_(0) and *W*_*i*_(0). The values of *σ*_*i*_, $i\in {{{{{{{\mathcal{I}}}}}}}}$, are then assumed to remain constant at successive time steps of the simulation, from time 1 to *M*. Finally, we set the floor of the probability of default for each node *i* as $PD{M}_{i}^{floor}=0.00021$, which is the upper end of the AAA default probability bracket within the internal credit rating methodology used by Credit Suisse^49^. In the sequel, we denote the available governmental investment actions by

$$ < {{{{{{{\rm{node}}}}}}}} > \,@ \, < \,{{\mbox{capital investment as a tenth of a percent of the total asset}}}\,W > ,$$

with the convention that an action that considers all nodes is indicated with <node > = 0. For example, 8@05 means an investment of 50 *bp* *W*_8_ or 0.5% *W*_8_ in node 8, while 0@15 stands for an investment of 1.5% *W*_*i*_ in each node $i\in {{{{{{{\mathcal{I}}}}}}}}\setminus {{{{{{{{\mathcal{I}}}}}}}}}_{def}$.

The common theme is that adding external resources makes the network more resilient, but such resources can be lost in a subsequent default, which creates a trade-off for the decision-maker. The optimal policy that balances this trade-off and minimises the overall expected taxpayers’ loss is an optimal solution to our MDP, and is analysed in the forthcoming two studies.

### Case study 1: KK network

**Fig. 1:**
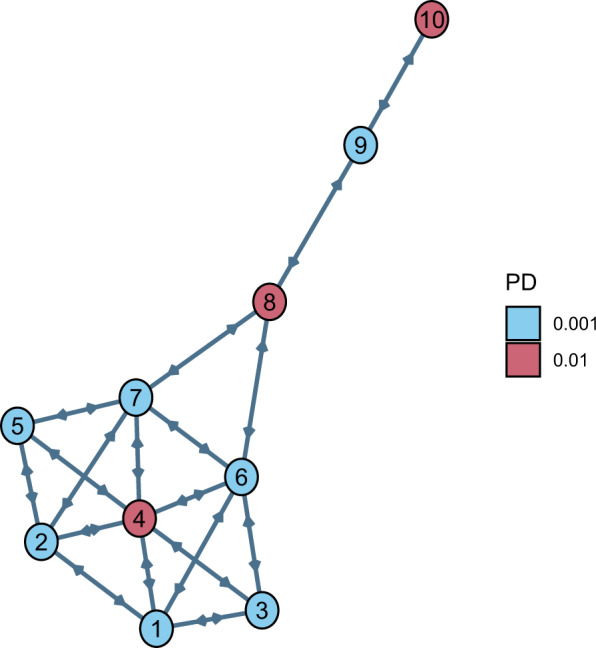
Krackhardt Kite (KK) network. The KK network is used to assess how bailout decisions are influenced by node centrality. The ten nodes of the graph in ${{{{{{{\mathcal{I}}}}}}}}=\{1,\ldots,10\}$ represent financial institutions, which are identical apart from their probabilities of default (PD) at time 0, which are *PD*_*i*_(0) = 0.01, for *i* ∈ {4, 8, 10}, and *PD*_*i*_(0) = 0.001, for $i\in {{{{{{{\mathcal{I}}}}}}}}\setminus \{4,8,10\}$. They all have a normalised total asset *W*_*i*_(0) = 100 and capital *E*_*i*_(0) = 3. The edges between nodes, representing claims between financial institutions, are oriented and homogeneous, assuming the value *w*_*ij*_ = 1, for all $i\,\ne\, j\in {{{{{{{\mathcal{I}}}}}}}}$.

This study concerns a network with homogeneous nodes organised as the Krackhardt kite (KK) graph (Fig. 1, see also ref. 50), which is referred to as the KK network. The main reason for choosing the KK graph as an underlying network is to primarily assess whether our algorithm can distinguish between central nodes and peripheral ones. In particular, we use the network characteristics in terms of the centrality of nodes 4, 8 and 10 (see Fig. 1), to investigate how bailout decisions depend on the nodes’ position in the network. However, we will also investigate additional hypotheses and reach important financial conclusions on bailout decision making.

In this case study, all the nodes (banks or other financial institutions) have total asset *W*_*i*_(0) = 100 and capital *E*_*i*_(0) = 3. As shown in Fig. 1, the nodes in red colour have probability of default *PD*_*i*_(0) = 0.01, for *i* ∈ {4, 8, 10}, while the others have *PD*_*i*_(0) = 0.001, for $i\in {{{{{{{\mathcal{I}}}}}}}}\setminus \{4,8,10\}$. The edges between nodes are oriented and homogeneous, assuming the value *w*_*ij*_ = 1, for all $i \,\ne\, j\in {{{{{{{\mathcal{I}}}}}}}}$. For the sake of this case study, we restrict the potential investment amounts in each node *i* to be: 0, 0.5% *W*_*i*_, 1% *W*_*i*_, 1.5% *W*_*i*_ or 2% *W*_*i*_. Furthermore, the government can choose at each time step to invest in the single nodes 4, 8, 10 or in all nodes.

**Fig. 2:**
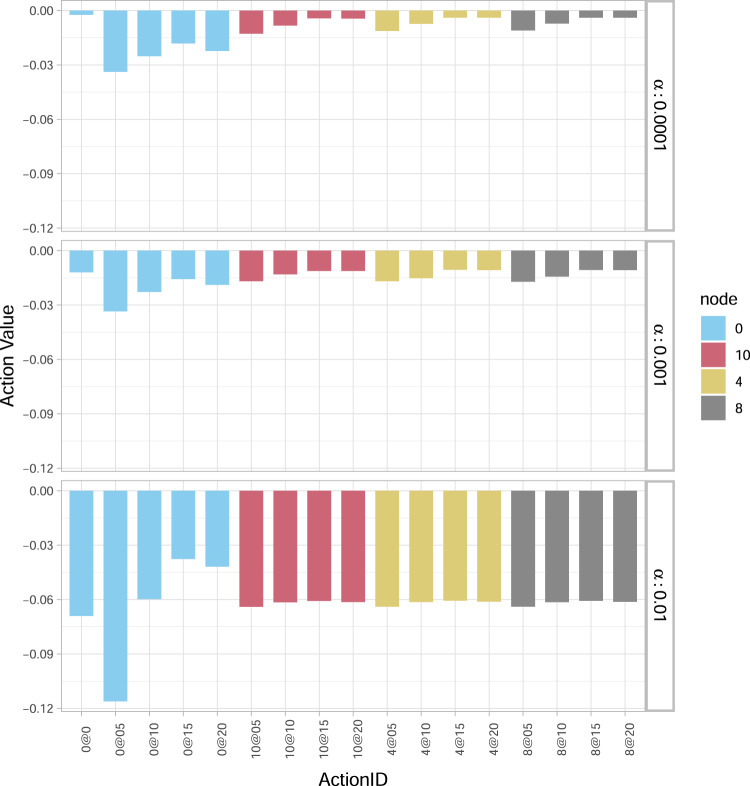
Optimal action value function Q_*_ for the Krackhardt Kite (KK) network. The optimal action value function Q_*_(*s*_0_, *a*_0_) at time 0 for different actions *a*_0_ (a government investment of 0, 0.5, 1, 1.5 or 2 in the equity of the nodes) and values of percentage wealth loss upon default *α* (0.0001, 0.001 and 0.01). In the legend, the colours identify the nodes {0, 10, 4, 8} in the figure (0 represents all nodes). On the *x* axis, the ActionID 0@0 means no investment, 0@05 means investing 0.5 in all the nodes, 10@05 means investing 0.5 in node 10, etc. For a small value of *α* = 0.0001, the best action is not to invest (0@0). As *α* increases, so does the convenience of investing more capital. For *α* = 0.01 the best action (corresponding to smallest loss) is to invest 1.5 in all the nodes (0@15). It is never convenient to invest the maximum amount of capital (0@20), while investing 0.5 in all the nodes (0@05) is the worst action for all values of *α*, as the additional capital is not enough to strengthen the network and it is at risk following potential defaults.

The optimal action value function Q_*_(*s*_0_, *a*_0_) at time 0 is illustrated in Fig. 2 for three scenarios of percentages of wealth loss upon default. In particular, for a “relatively low” *α* = 0.0001, the best action (minimising losses) is not to invest in any bank (0@0). Moving from the top to the bottom panel (as *α* increases) the option not to invest becomes more and more costly to the system. For a “relatively intermediate” *α* = 0.001, not investing is roughly equally favourable to investments in single financial institutions, while for a “relatively high” *α* = 0.01, the best action becomes to invest 1.5% *W*_*i*_ in all financial institutions (0@15). It is also interesting to note that (see Fig. 2) irrespective of the *α*-value: (i) investing the maximum amount of 2% *W*_*i*_ in all banks (0@20) is never the best choice; (ii) providing the minimum capital (0@05) is always the worst choice, as the additional investment is too small to make them resilient, but still increases the funds at risk in case of default. The sensitivity of the optimal policy with respect to *α* will be further examined in more detail also in our next (more realistic) case study.

**Fig. 3:**
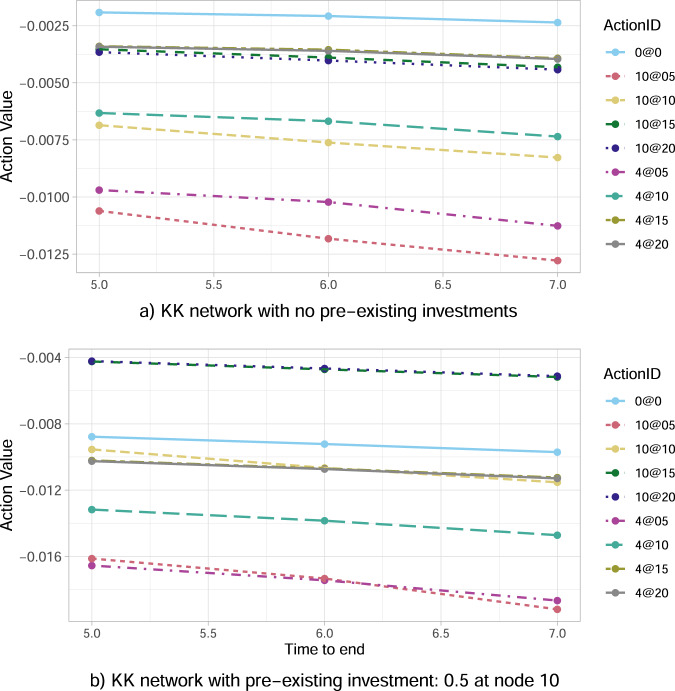
Optimal action value function Q_*_ for the Krackhardt Kite (KK) network as a function of time to the end of crisis. The results are obtained for the percentage *α* = 0.0001 of wealth loss upon default, and focus on nodes 4 (central node) and 10 (peripheral node). In the legend, the ActionID 0@0 means no investment, 0@05 means investing 0.5 in all the nodes, 10@05 means investing 0.5 in node 10, etc. **a** The algorithm feels the network structure and suggests to invest in node 4 (leading to smaller loss) rather than node 10. **b** In case the government had previously invested in node 10, the government needs to protect its investment by optimally risking an additional investment in node 10.

Fixing the percentage of wealth loss upon default at *α* = 0.0001, we now focus our analysis on the central node 4, representing a financial institution with multiple links and interconnections with its peers, versus the peripheral node 10, representing a relatively isolated financial institution linked only with one other (see Fig. 1). The results in Fig. 3a conclude that investing in the central node 4 is always better than in the peripheral node 10 for the same amount of capital and all such choices. Thus, our algorithm indeed shows a clear preference in central rather than peripheral node investments.

However, the results change when the government had already invested even the minimum possible amount of 0.5% *W*_10_ in the peripheral node 10. In this case, Fig. 3b with *J*_10_(0) = 0.5 concludes that a substantial additional investment in bank 10 (namely, 10@15 or 10@20) largely outperforms any other strategy—including not investing at all, and all types of investments in the central node 4. Such a result indicates that the optimal strategy for the government is therefore to keep investing (sufficiently high amounts of capital) in node 10, aiming at saving this already invested capital *J*_10_(0) = 0.5. This governmental tendency to provide capital to distressed banks if they had already invested in them creates moral hazard, as the bank could act haphazardly relying on the implicit government guarantee. The fact that bailouts create moral hazard has been emphasised extensively in the financial and economic literature, both theoretically and empirically (see e.g., refs. 51–54). Moreover, given that the assumed *J*_10_(0) = 0.5 is the worst amongst all possible investments in a single node at time 0 (see both Fig. 2 and Fig. 3a), the suggested optimal, additional, significantly large investment in the peripheral node 10, could be also viewed as an eventual strengthening of the originally weak investment of 0.5% *W*_10_.

Lastly, we perform a sensitivity analysis of the optimal action versus the duration of crisis. Irrespective of the type of investment, the results in Fig. 3a show that the optimal action value function Q_*_ decreases in absolute value (i.e. size of losses decreases) as the time to the end of the episode *M*−*t* decreases. The main reason is that each node of the network is unstable with an associated probability of default per unit time, hence the shorter the time horizon the lower the expected losses. Furthermore, the contagion has less time to propagate, which explains also why the node’s position in the network becomes less and less relevant.

### Case study 2: EBA network

**Fig. 4:**
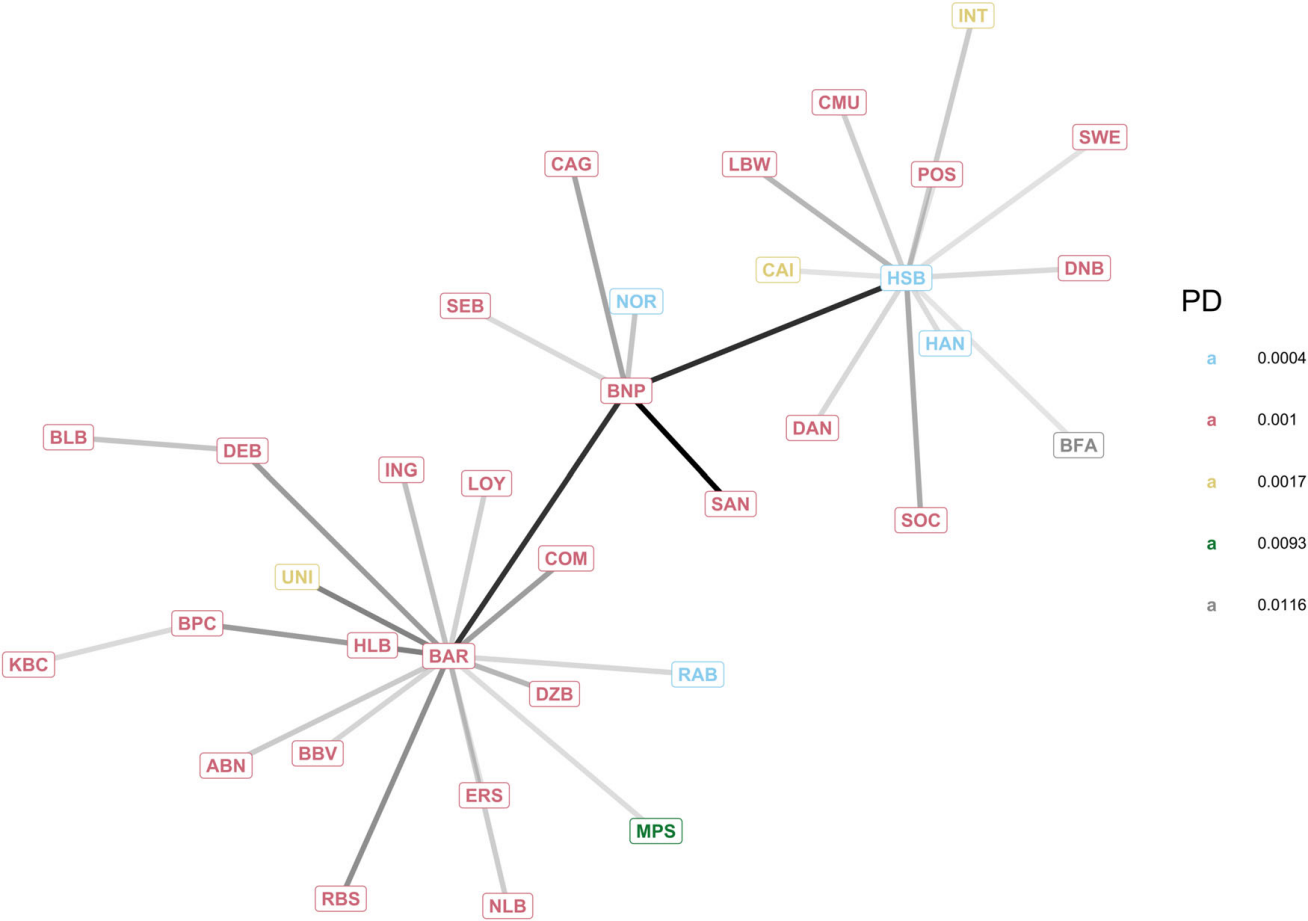
Maximum spanning tree of the European Banking Authority (EBA) network. The network of the European Union’s Global Systemically Important Institutions (GSII) has been reconstructed from aggregated data available at the EBA website^55^. Each node represents a financial institution (see Table 1), its colour represents its probability of default (PD), and darker edges identify stronger exposures.

After having considered a small synthetic graph in the first case study, we now study a network of the European global systemically important institutions (GSII, see Table 1) obtained from the data provided by the European Banking Authority^55^, which is referred to as the EBA network. Note that, the original data do not contain the complete bilateral network of exposures, as this is considered business-sensitive information. While the specific exposure between two banks is unknown, the aggregated credit exposure of a bank versus other financial institutions is provided. For each bank *i* in the set ${{{{{{{\mathcal{I}}}}}}}}$ of the European GSII, we have its total inter-bank asset ${\sum }_{j\in {{{{{{{\mathcal{I}}}}}}}}}{w}_{ij}$ and liability ${\sum }_{j\in {{{{{{{\mathcal{I}}}}}}}}}{w}_{ji}$, which can be used to reconstruct a network that satisfies the constrains (see, e.g. algorithms described in refs. 37, 42). The reconstructed network (see Fig. 4) can be different but has similar characteristics to the actual network of bilateral exposures. The values of total asset *W*_*i*_(0) and capital *E*_*i*_(0) at time 0 used for each financial institution *i* are reported in Table 1. The probabilities of default are derived using data from the credit rating agency Fitch^56^ and show that the nodes with the higher probability of default are Monte dei Paschi di Siena (MPS) and BFA (see both Table 1 and Fig. 4).

**Table 1:** European Union’s Global Systemically Important Institutions (GSII)

SYMBOL	W	E	PD	BANK
BFA	235	12	0.0116	BFA
MPS	201	7	0.0093	Monte dei Paschi di Siena
UNI	1034	45	0.0017	Unicredit
INT	696	38	0.0017	Intesa Sanpaolo
CAI	377	19	0.0017	La Caixa
BNP	2253	70	0.001	BNP Paribas
BAR	1940	59	0.001	Barclays
CAG	1723	71	0.001	Credit Agricole
DEB	1659	63	0.001	Deutsche Bank
SAN	1456	64	0.001	Santander
RBS	1411	51	0.001	RBS
SOC	1409	47	0.001	Societe Generale
BPC	1337	50	0.001	BPCE
ING	1164	41	0.001	ING
LOY	1107	46	0.001	Lloyds
BBV	723	42	0.001	BBVA
CMU	695	37	0.001	Credit Mutuel
COM	656	25	0.001	Commerzbank
DAN	494	19	0.001	Danske Bank
ABN	421	16	0.001	ABN Amro
DZB	356	13	0.001	DZ Bank
DNB	332	15	0.001	DNB
SEB	310	13	0.001	SEB
LBW	290	13	0.001	LBBW
BLB	275	10	0.001	Bayern LB
SWE	249	10	0.001	Swedbank
KBC	232	14	0.001	KBC
POS	223	7	0.001	Banque Postale
ERS	219	11	0.001	Erste Group
NLB	216	7	0.001	NordLB
HLB	199	8	0.001	Helaba
HSB	2680	117	0.0004	HSBC
RAB	728	34	0.0004	Rabobank
NOR	655	25	0.0004	Nordea
HAN	334	11	0.0004	Handelsbanken

The total asset (W) and Tier 1 capital (E) are expressed in billions of EUR. The data are from the European Banking Authority (EBA)^55^ and are relative to the end of 2014. The probabilities of default have been derived using data from the Fitch credit rating agency^56^.

To facilitate our analysis, we firstly pretend that the European Union (including the UK) is a fiscal union with a single regulator (“government”) that is accountable to all European taxpayers. Then, we consider any individual states’ investments in banks prior to 2014 as “private” investments, hence we set the initial regulator investments to be *J*_*i*_(0) = 0 for all $i\in {{{{{{{\mathcal{I}}}}}}}}$. For the sake of this case study, we restrict the potential investment amounts to inject in each financial institution *i* to be: 0, 0.5% *W*_*i*_, 1% *W*_*i*_, 1.5% *W*_*i*_, 2% *W*_*i*_, 2.5% *W*_*i*_ or 3% *W*_*i*_. Furthermore, we assume that the government can choose at each time step *t* to invest in all the nodes that are considered “risky” at that time, defined as each financial institution $i\in {{{{{{{\mathcal{I}}}}}}}}\setminus {{{{{{{{\mathcal{I}}}}}}}}}_{def}$ with *PD*_*i*_ > 0.009, according to our (arbitrarily) chosen threshold. In this case study, the notation 0*@*05 thus indicates an investment of 0.5% *W*_*i*_ in each risky node $i\in {{{{{{{\mathcal{I}}}}}}}}\setminus {{{{{{{{\mathcal{I}}}}}}}}}_{def}$.

**Fig. 5:**
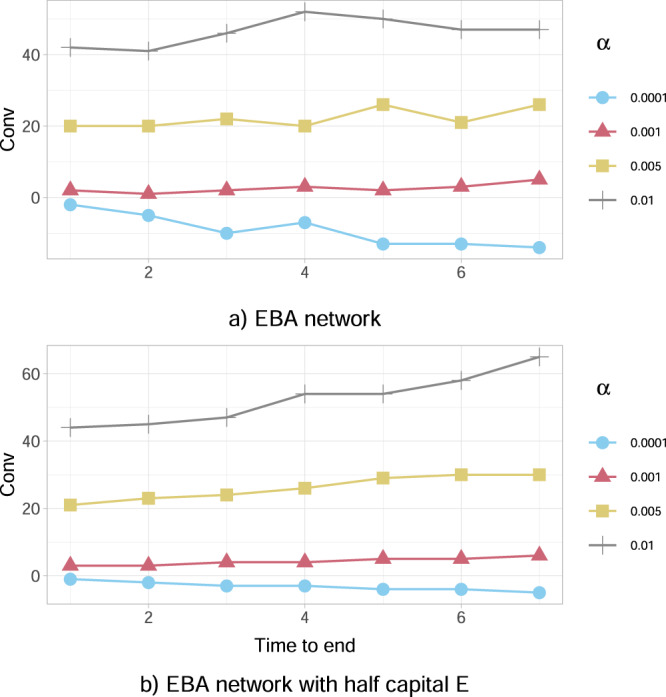
The convenience to intervene Conv for the European Banking Authority (EBA) network as a function of time to the end of crisis. **a** The Conv (in millions of EUR) defined in (23) is positive for higher percentages of wealth loss upon default (*α* = 0.01, 0.005, 0.001), thus investing is a favourable action. Conversely, Conv < 0 for smaller *α* (*α* = 0.0001), implying that it is not convenient for the government to invest. Conv tends to be an increasing function of the time to the end of the crisis when positive, and a decreasing function when negative. **b** A severely distressed version of the network, where the banks' capital *E*_*i*_(0) has been artificially halved (all other characteristics are the same). We observe that such a distress has the effect of increasing Conv for each value of *α*.

For a detailed quantitative and qualitative analysis, we rely on the convenience measure Conv for the government, defined in (23), to intervene with equity investments and we analyse the system for four different percentages *α* ∈ {0.0001, 0.001, 0.005, 0.01} of wealth loss upon default. We observe from Fig. 5a that, we have a convenience Conv > 0 for higher percentages of wealth loss upon default (*α* = 0.01, 0.005 and 0.001), thus investing is a favourable action, while Conv < 0 for smaller *α* (*α* = 0.0001), implying that it is not convenient for the government to invest. This is consistent with our first case study using the KK network (previous subsection), as investing an amount of capital is convenient only for relatively high values of *α*, in order to make the network sufficiently resilient.

**Fig. 6:**
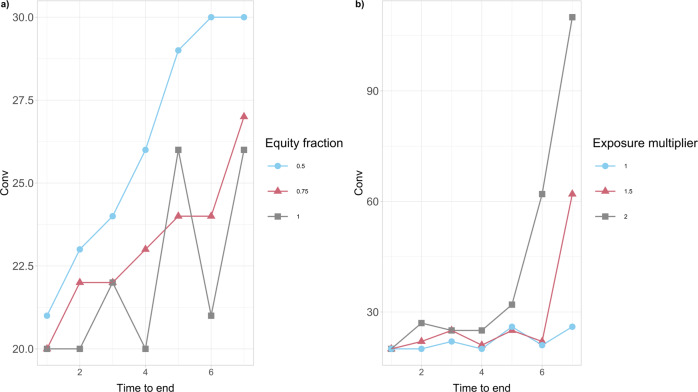
The convenience to intervene Conv for the European Banking Authority (EBA) network as a function of the time until the end of crisis. The values of Conv (in millions of EUR) are obtained for the percentage *α* = 0.005 of wealth loss upon default. **a** We consider different percentages (50%, 75%, 100%) of the initial capital *E*_*i*_(0) available to financial institutions *i* at time 0. Conv tends to increase on average as the financial institutions’ initial capital *E*_*i*_(0) decreases. It is also interesting to note that, this convenience intensifies for larger lengths of time until the end of the crisis. **b** We consider different multipliers (1, 1.5, 2) of the bilateral credit exposures *w*_*ij*_ of financial institutions *i* to the default of *j* at time 0. Conv increases as the *w*_*ij*_’s increase and the impact of longer crisis duration on Conv is massive.
- It is also clear from the results in Fig. 6b that the convenience to intervene increases as the bilateral credit exposures *w*_*ij*_ between financial institutions across the whole network increase. It is interesting to further observe that the impact of longer crisis duration on the convenience to intervene is massive.

A sensitivity analysis of the convenience to intervene for the government is further examined versus the duration of crisis, the initial capital and the credit exposures of financial institutions:

- The results in Fig. 5a further conclude that the convenience to intervene tends to be an increasing function of the time to the end of the episode *M*−*t* when Conv > 0 and a decreasing function when Conv < 0. This implies that the convenience to intervene or not, weakens (decreases in absolute value) as we approach the end of the crisis. Interestingly though, it appears that the nature of the action does not change with time, since the function Conv does not change sign.
- Our results in Fig. 5b further show that the convenience to intervene Conv is dependent on the banks’ resilience, expressed via the initial capital *E*_*i*_(0) of each bank *i*. In particular, this severely distressed version of the network, where the value of *E*_*i*_(0) has been artificially halved compared with the original case study presented in Fig. 5a, has the effect of increasing the convenience Conv for the government to intervene for each value of *α*. A more thorough analysis in Fig. 6a further reveals that the convenience to intervene increases on average as the financial institutions’ initial capital *E*_*i*_(0) decreases, for all duration lengths of the crisis. It is interesting to also note that, this convenience intensifies significantly for larger lengths of time until the end of the crisis.

**Fig. 7:**
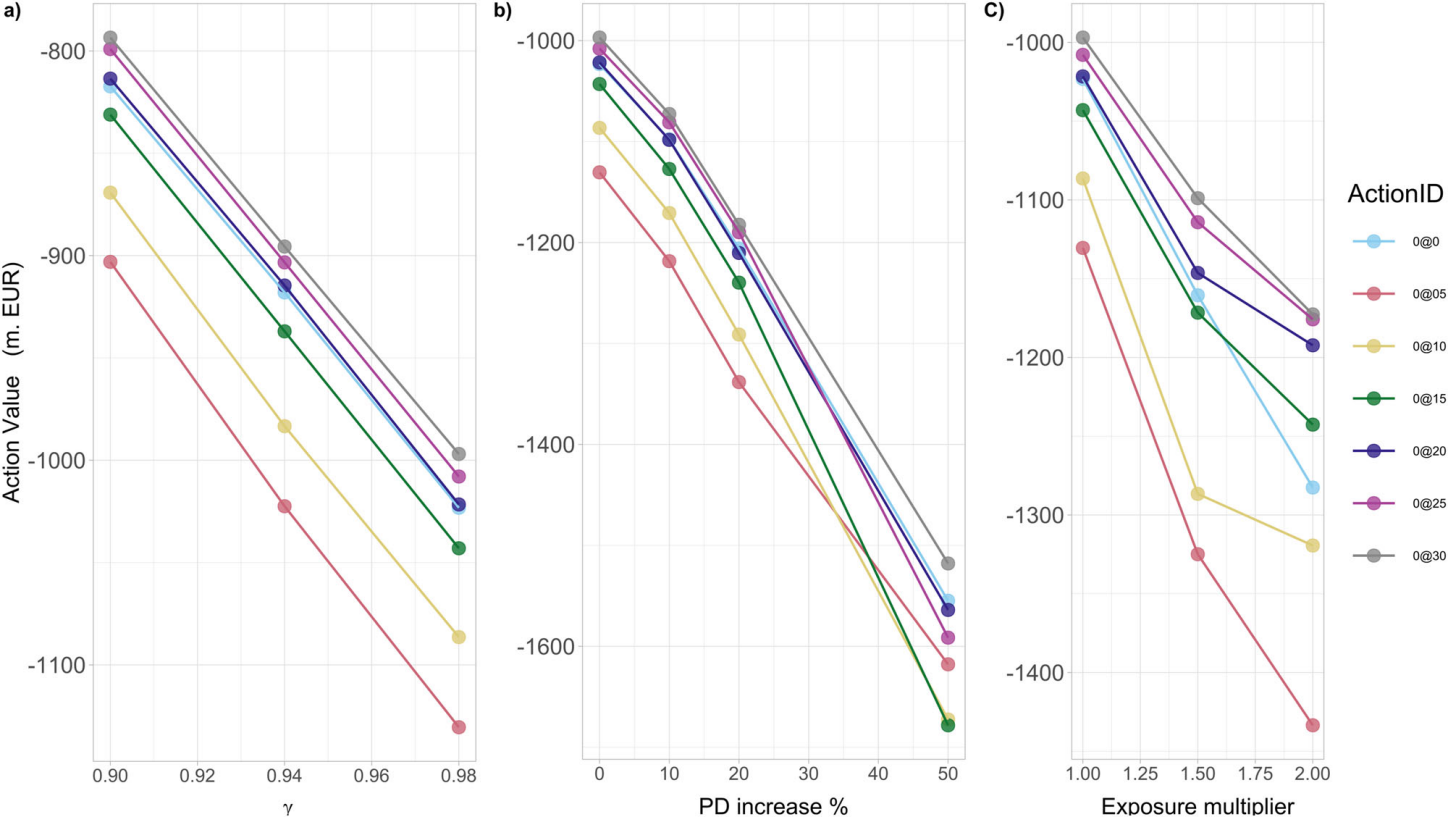
The optimal action value Q_*_(*s*_0_, *a*_0_) for the European Banking Authority (EBA) network at time 0. In the legend, ActionID 0@0 means no investment, 0@05 means investing 0.5 in all the nodes, etc. The results are obtained for the percentage *α* = 0.005 of wealth loss upon default. **a** As a function of discount factor *γ*, Q_*_(*s*_0_, *a*_0_) decreases, for all potential actions *a*_0_, as *γ* increases. **b** As a function of the percentage increase of the probabilities of default *PD*_*i*_(0) of financial institutions *i* at time 0, Q_*_(*s*_0_, *a*_0_) decreases, for all potential actions *a*_0_, as *PD*_*i*_(0) increase. **c** As a function of the magnitude of multiplier of the bilateral credit exposures *w*_*ij*_ of financial institutions *i* to the default of *j*, Q_*_(*s*_0_, *a*_0_) decreases, for all potential actions *a*_0_, as *w*_*ij*_ increase.
- Our results in Fig. 7b then show that the optimal action value function Q_*_(*s*_0_,*a*_0_) at time 0 decreases (taxpayers’ losses increase in absolute value) for all potential actions, with increasing probabilities of default. Two interesting features appearing are: (i) the initially narrowly optimal action (0@30) becomes clearly optimal as the probabilities of default increase; (ii) the worst possible action, namely the one to avoid, changes from the smallest possible investment of 0.5%*W*_*i*_ to larger investments of 1.0%*W*_*i*_, 1.5%*W*_*i*_ in all risky financial institutions *i*. That is, even these medium size additional investments are not enough to make them resilient, but still significantly increase the funds at risk in case of default.
- Our results in Fig. 7c also show that the optimal action value function Q_*_(*s*_0_, *a*_0_) at time 0 decreases for all potential actions, with increasing bilateral credit exposures *w*_*ij*_ between financial institutions across the whole network. We also note that the difference in the performance of the optimal investment of a large amount (0@30) and non investing at all, increases with greater credit exposures amongst institutions.
- It has already been confirmed by both case studies under consideration (KK and EBA network subsections), that as the percentage *α* of potential wealth loss upon default increases, the inaction (no investments) becomes less convenient for the government. We now aim to explore further the transition between the scenarios when it is convenient and not convenient for a regulator to intervene, by studying the optimal action values Q_*_(*s*_0_, *a*_0_) at time 0 with respect to changes in *α*. Our results in Fig. 8a conclude that: (i) there exists a critical *α*_*c*_ ≈ 0.00079 that splits the parameter space of *α*-values into two “wealth loss regimes” of high/low values, reflecting governmental action/inaction, respectively; (ii) the optimal action at time 0 changes drastically (non-smoothly) from a do not invest anything policy for *α* just below *α*_*c*_ to an invest the maximum amount of 3.0%*W*_*i*_ in all risky institutions *i* (0@30) policy for *α* just above *α*_*c*_. Notice that, these actions are in fact the two extremes. This is an interesting result as one might have expected a smoother transition between optimal actions as *α* increases.

A sensitivity analysis of the governmental optimal action is also examined versus the discount factor, the financial institutions’ probabilities of default and credit exposures, the percentage of wealth loss upon default and the initial capital:

- Our analysis in Fig. 7a shows clearly that the optimal action value function Q_*_(*s*_0_, *a*_0_) at time 0 decreases for all potential actions as the discount factor *γ* increases. That is, as the future losses become more relevant (from the governmental point of view), the expected systemic losses increase in absolute value, while the optimal action does not change qualitatively.

**Fig. 8:**
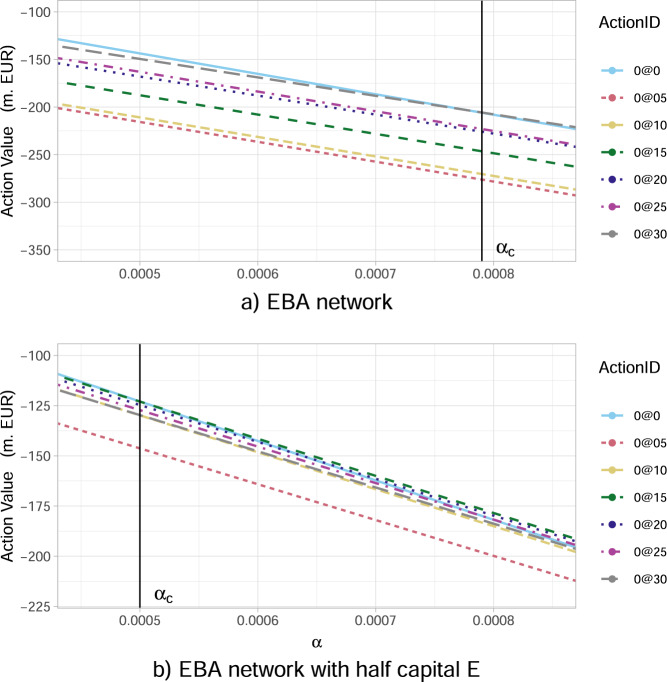
The optimal action value Q_*_(*s*_0_, *a*_0_) for the European Banking Authority (EBA) network at time 0 as a function of the percentage *α* of wealth loss upon default. In the legend, the ActionID 0@0 means no investment, 0@05 means investing 0.5 in all the nodes, etc. **a** There is a critical value of *α* given by *α*_*c*_ ≈ 0.00079, beyond which the inaction of the government is no longer optimal. In particular, for *α* just above the critical *α*_*c*_, the best action becomes the investment of 3.0%*W*_*i*_ in all risky financial institutions *i* (0@30). **b** A severely distressed version of the network, where the banks' capital *E*_*i*_(0) has been artificially halved (all other characteristics are the same). We observe that such a distress affects Q_*_(*s*_0_, *a*_0_) at time 0 and the value *α*_*c*_ at which a regulatory intervention becomes favourable is lower, namely *α*_*c*_ ≈ 0.0005. The optimal action becomes the investment of 1.5% *W*_*i*_ in all risky financial institutions *i* (0@15).
- We then conclude from our results in Fig. 8b, which is a severely distressed version of the original network (presented in Fig. 8a), where the financial institution *i*’s capital *E*_*i*_(0) has been artificially halved, that the optimal action value function Q_*_(*s*_0_, *a*_0_) at time 0 changes significantly both quantitatively and qualitatively. Interestingly, the optimal action for *α* > *α*_*c*_ becomes the considerably decreased investment of 1.5% *W*_*i*_ in all risky financial institutions *i* (0@15), compared to the original network (see Fig. 8a). A more thorough analysis in Fig. 9 reveals that, as the initial capital *E*_*i*_(0) decreases, the optimal investment amount indeed decreases as well. In particular, we observe that the investment of 3.0%*W*_*i*_ in all risky financial institutions *i*, becomes 2.5%*W*_*i*_ when the capital decreases by 25% and 1.5%*W*_*i*_ when the capital decreases by 50%. Furthermore, our results in Fig. 9 reveal that the universally (for all *E*_*i*_(0)) worst action is to invest the lower amount of 0.5%*W*_*i*_ in all risky financial institutions *i*, as such an additional investment is too small to make them resilient, but still increases the funds at risk in case of default.

**Fig. 9:**
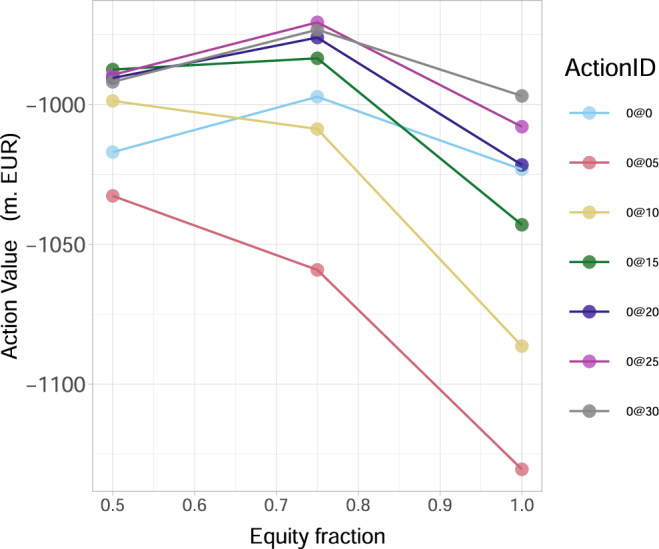
The optimal action value Q_*_(*s*_0_, *a*_0_) for the European Banking Authority (EBA) network at time 0 as a function of the percentage of initial capital *E*_*i*_(0) available to financial institutions *i*. In the legend, the ActionID 0@0 means no investment, 0@05 means investing 0.5 in all the nodes, etc. The results are obtained for the percentage *α* = 0.005 of wealth loss upon default and show that the best action a^*^ amongst *a*_0_’s (corresponding to the highest Q_*_(*s*_0_, *a*_0_) value) decreases (0@30 → 0@25 → 0@15) as *E*_*i*_(0) decrease.

Lastly, we perform a sensitivity analysis of the aforementioned wealth loss regimes versus the financial institutions’ initial capital. We can conclude from our results in Fig. 8b that, as the financial institution *i*’s capital *E*_*i*_(0) decreases, the value *α*_*c*_ separating the two wealth loss regimes of unfavourable and favourable regulatory interventions, is significantly lower *α*_*c*_ ≈ 0.0005. Namely, the government is willing to intervene for much lower percentages of wealth loss.

## Discussion

The main theoretical contribution of this paper is twofold. On the modelling side, we propose a framework with a dynamic network of financial institutions, which allows governments or regulatory bodies to assess and quantify bank bailout decisions; we further show how these decisions can be cast into actions in a Markov Decision Process (MDP), where the states of the MDP are defined in terms of the underlying network of financial exposures and the MDP dynamics is derived from the network dynamics. On the optimisation side, in order to identify the optimal governmental investment policy from the taxpayers’ standpoint, for each state of the financial institutions network at each time *t*, we develop a methodology involving artificial intelligence techniques that learn the optimal bailout actions to minimise the taxpayers’ losses (for details refer to the Methods section). This methodology goes beyond standard theory and overcomes several technical hurdles, which could also have a significant impact on tackling stochastic control problems in dynamic networks with few reward signals.

In the implementation of our framework and methodology in two case studies, it is evident from our analysis that the loss for the taxpayers, as a fraction *α* of the financial institution’s total assets upon default, plays a central role in systemic risk modelling. In our main case study, which uses the data relative to the European global systemically important institutions, we find that governmental interventions do not improve the expected loss of the financial network if *α* < *α*_*c*_, for some critical threshold value *α*_*c*_, which is determined by the network’s characteristics and is decreasing as the distress of the network increases. We also find that the convenience to intervene increases for longer crisis time horizons, smaller banks’ resilience (equity) and higher bank bilateral credit exposures. Moreover, using a well-known small graph (the Krackhardt kite network) as a simplified case study, we find that even though investing in central nodes is a priori more favourable, the government should optimally keep investing in a node if it had already invested in it in the past, even if that node is not a central one in the network. The government needs to evaluate carefully a potential investment, since the rescued financial institution could increase its risky investments knowing that it would be bailed-out in case it became distressed again, thus leading to moral hazard.

The modelling and optimisation methods in this paper can be extended in several directions. The decision maker could introduce a second layer of stochasticity to the model, driving the market conditions after the end of the crisis. This would allow the additional optimisation of the sale timing and price of the acquired shares of rescued (surviving) financial institutions, in view of maximising the taxpayers’ profits or minimising their losses from these investments. Another possible extension could be the consideration of an action-dependent end of the crisis time *M*_*t*_ ≔ *M*( *J*_1_(*t*),…, *J*_*N*_(*t*)), whereby large bailout decisions could shorten the duration of the crisis. Such extensions would require the use of other methodologies, which could lead to interesting future research projects.

## Methods

### Value function approximation

In order to solve our MDP using our variation of a Fitted Value Iteration algorithm, we need a parametric representation of the optimal value function V_*_(*s*_*t*_). In our case, we see from (20) that V_*_(*s*_*t*_) is minus the minimum expected cumulative loss when starting from state *s*_*t*_ (i.e. the maximum expected discounted cumulative reward from state *s*_*t*_), incurred between time *t* and the end of the episode at time *M*.

The potential for additional losses in the financial network increases with the number of (surviving) nodes and number of residual steps *m* ≔ *M*−*t* until the end of the crisis. It is thus natural to try to approximate V_*_(*s*_*t*_) by a (weighted) sum of the expected direct loss contributions due to each individual node at each of the remaining *m* time steps (cf. optimisation criterion in (20)).

We estimate these expectations using an approximation of each node’s probability of default and overall wealth at all remaining time steps. To do so, our methodology prescribes to firstly use the current probabilities of default for each step to approximate the expected future impact of defaults on each node *i* at the next time step. These expected impacts are then used as input in order to estimate the (expected) future wealth, equity and obtain an updated approximate probability of default for each node *i* at that next time step, which closes the loop. By following this procedure, we are then able to obtain an approximation *Z*_*ik*_(*s*_*t*_) of the expected direct loss contribution of each individual node $i\in {{{{{{{\mathcal{I}}}}}}}}\setminus {{{{{{{{\mathcal{I}}}}}}}}}_{def}(t)$ at each remaining time step *k* ∈ {1,…,*m*}, for any arbitrary policy *π*.

In order to obtain the matrix $\overline{{{{{{{{\bf{Z}}}}}}}}}: \!\!=({\overline{Z}}_{ik}({s}_{t}))$ that will be used to define our value function approximation, we take into account potential government interventions to minimise the taxpayers’ losses. This involves a sequential optimisation whereby, at each remaining step *k*, the government chooses the action ${\overline{a}}_{t+k-1}$ that minimises the sum of the contributions *Z*_*ik*_, assuming no intervention at future steps and using the already chosen actions for previous steps. Further mathematical details of our choice of $\overline{{{{{{{{\bf{Z}}}}}}}}}$ in terms of the characteristics of the network are given in the following Value function parametrisation subsection.

Our ansatz for the parametric representation ${\overline{V}}_{*}({s}_{t},\beta )$ of V_*_(*s*_*t*_) is that it is given by a linear combination of the elements ${\overline{Z}}_{ik}$, in which the coefficients (weights) *β* are arranged in a matrix that can change with time, i.e. *β* ≡ *β*_*t*_ ≔ (*β*_*ik*_(*t*)). Namely,

27 $${\overline{V}}_{*}({s}_{t},{\beta }_{t}):=-\mathop{\sum}\limits_{i,k}{\beta }_{ik}(t){\overline{Z}}_{ik}({s}_{t}),\qquad {{{{{{{\rm{for}}}}}}}}\,i\in {{{{{{{\mathcal{I}}}}}}}}\setminus {{{{{{{{\mathcal{I}}}}}}}}}_{def}(t),\,k\in \{1,\ldots,m\}.$$

### Value function parametrisation

In this subsection, we detail our choice for $({\overline{Z}}_{ik}({s}_{t}))$ used in our ansatz for the value function approximation ${\overline{V}}_{*}({s}_{t},\beta )$ in (27), with node $i\in {{{{{{{\mathcal{I}}}}}}}}\setminus {{{{{{{{\mathcal{I}}}}}}}}}_{def}(t)$ and artificial step *k*∈{1,…,*m* = *M*−*t*}, where each *k* corresponds to the time step *t* + *k*−1. The last step *k* = *m*, therefore, refers to time *M* −1, namely the last step away from the end of the episode. In the following, we denote by *W*_*i*_, *E*_*i*_ and *J*_*i*_ the current levels of wealth, equity and cumulative investment at time *t*.

We introduce the auxiliary matrix **Z**, with elements *Z*_*ik*_(*s*_*t*_;*a*_*t*_,…,*a*_*t*+*k*−1_) representing our approximation of the expected direct loss contribution due to the default of node *i* at time *t* + *k*−1, taking into account the government actions *a*_*t*+*j*−1_ at times *t* + *j*−1, for all *j* = 1,…,*k*. That is, we approximate the expected values of the summands involved in the optimisation criterion (20) via the terms

$${Z}_{ik}: \!\!=\left\{\begin{array}{ll}P{D}_{ik}{L}_{ik},\quad \hfill &{{{{{{{\rm{if}}}}}}}}\,k=1;\\ P{D}_{ik}{\gamma }^{k-1}{L}_{ik}\mathop{\prod }\nolimits_{r=1}^{k-1}(1-P{D}_{ir}),\quad &{{{{{{{\rm{if}}}}}}}}\,k > 1,\end{array}\right.$$

where, the approximate probability of default of each node *i* at time *t* + *k*−1 is given by a value *PD*_*ik*_ and the approximate taxpayers’ loss by a value *L*_*ik*_. Note that in this approximation, for a step *k* > 1, a node *i* can contribute to the expected loss, only if it has not defaulted in the previous steps; hence, the presence of the survival probabilities 1−*PD*_*ir*_, for all *r* ∈ {1,…,*k*−1}.

To be more precise with the above approximations, we begin by approximating each node’s probability of default (cf. its original definition in (13)) via

$$P{D}_{ik}: \!\!=\max \left\{PDM({W}_{i}+{J}_{ik}-{I}_{ik},{E}_{i}+{J}_{ik}-{I}_{ik},{\mu }_{i},{\sigma }_{i}),PD{M}_{i}^{floor}\right\},$$

which takes into account the potential cumulative investment *J*_*ik*_ from the government and our approximation of the expected cumulative impact *I*_*ik*_ on node *i* up to time *t* + *k*−1. On one hand, the cumulative investment *J*_*ik*_ in node *i* is a function of the actions (*a*_*t*_,…,*a*_*t*+*k*−1_) that the government can take between *t* and *t* + *k*−1 and is independently defined (via its standard definition) by

$${J}_{ik}({a}_{t},\ldots,{a}_{t+k-1}): \!\!=\mathop{\sum }\limits_{r=1}^{k}\Delta {J}_{i}^{a}(t+r-1).$$

On the other hand, we approximate the expected cumulative impact *of I*_*ik*_ via the approximated probability of defaults of all nodes $j\in H: \!\!={{{{{{{\mathcal{I}}}}}}}}\setminus ({{{{{{{{\mathcal{I}}}}}}}}}_{def}(t)\cup \{i\})$ at the previous time steps, which creates the desired approximation loop. Namely, (cf. its original definition in (15)) we obtain the approximation

$${I}_{ik}: \!\!=\left\{\begin{array}{ll}0\hfill \quad &{{{{{{{\rm{if}}}}}}}}\,k=1;\\ {\sum }_{j\in H}P{D}_{j1}{w}_{ij}\hfill \quad &{{{{{{{\rm{if}}}}}}}}\,k=2;\\ {I}_{ik-1}+{\sum }_{j\in H}P{D}_{jk-1}{w}_{ij}\mathop{\prod }\nolimits_{r=1}^{k-2}(1-P{D}_{jr})\quad &{{{{{{{\rm{if}}}}}}}}\,k > 2.\end{array}\right.$$

Consequently, *I*_*ik*_ is used as an input to approximate the taxpayers’ loss *L*_*ik*_ due to the default of node *i* at time *t* + *k*−1 (cf. its original definition in (16)) via

$${L}_{ik}: \!\!={\alpha }_{i}({W}_{i}+{J}_{ik}-{I}_{ik})+LG{D}_{i}({J}_{i}+{J}_{ik}).$$

At this point, we note that each *Z*_*ik*_ = *Z*_*ik*_(*s*_*t*_;*a*_*t*_,…,*a*_*t*+*k*−1_) depends on the actions (*a*_*t*_,…,*a*_*t*+*k*−1_) via the terms *J*_*ik*_(*a*_*t*_,…,*a*_*t*+*k*−1_) involved in both *PD*_*ik*_ and *L*_*ik*_. The only remaining task is therefore to provide an approximation for the actions (*a*_*t*_,…,*a*_*t*+*k*−1_) that optimise the aforementioned quantities *Z*_*ik*_ according to the objective in our stochastic control problem defined in (20). To that end, we define the total expected direct loss contribution *TL*(*s*_*t*_;*a*_*t*_,..,*a*_*M*−1_) as an approximation of the optimisation criterion in (20) for any arbitrary policy *π*, or equivalently any actions *a*_*t*_,..,*a*_*M*−1_. Namely, we aim at finding an approximation $({\overline{a}}_{t},\ldots,{\overline{a}}_{M-1})$ for the actions that minimise (over all nodes $i\in {{{{{{{\mathcal{I}}}}}}}}\setminus {{{{{{{{\mathcal{I}}}}}}}}}_{def}(t)$ in any of the remaining time steps *k*∈{1,…,*m*})

$$TL({s}_{t};{a}_{t},{a}_{t+1},..,{a}_{M-1}): \!\!=\mathop{\sum}\limits_{i,k}{Z}_{ik}({s}_{t};{a}_{t},{a}_{t+1},..,{a}_{t+k-1}).$$

To do so, we calculate each ${\overline{a}}_{t+j-1}$ sequentially for each step *j*∈{1,…,*m*} as follows:

$$	{\overline{a}}_{t}: \!\!=\mathop{{{{{{{{\rm{argmin}}}}}}}}}\limits_{{a}_{t}}TL\left({s}_{t};{a}_{t},{a}_{t+1}^{0},{a}_{t+2}^{0},..,{a}_{M-1}^{0}\right)\\ 	{\overline{a}}_{t+1}: \!\!=\mathop{{{{{{{{\rm{argmin}}}}}}}}}\limits_{{a}_{t+1}}TL\left({s}_{t};{\overline{a}}_{t},{a}_{t+1},{a}_{t+2}^{0},..,{a}_{M-1}^{0}\right)\\ \qquad\qquad\quad \vdots \\ 	{\overline{a}}_{M-1}: \!\!=\mathop{{{{{{{{\rm{argmin}}}}}}}}}\limits_{{a}_{M-1}}TL\left({s}_{t};{\overline{a}}_{t},{\overline{a}}_{t+1},\ldots,{\overline{a}}_{M-2},{a}_{M-1}\right),$$

with *a*^0^ denoting the action corresponding to no additional government investment. Then, the specific matrix $\overline{Z}=({\overline{Z}}_{ik}({s}_{t}))$ involved in our value function approximation ${\overline{V}}_{*}({s}_{t},\beta )$ in (27) is defined by

$${\overline{Z}}_{ik}({s}_{t}): \!\!={Z}_{ik}({s}_{t};{\overline{a}}_{t},\ldots,{\overline{a}}_{t+k-1}).$$

### Action value function approximation

Considering now the definition (24) of Q_*_(*s*_*t*_, *a*_*t*_) together with the approximation ${\overline{V}}_{*}({s}_{t},\beta )$ in (27) of V_*_(*s*_*t*_), we introduce ${\overline{Q}}_{*}({s}_{t},{a}_{t},{\beta }_{t+1})$ as the parametric representation of Q_*_(*s*_*t*_, *a*_*t*_), given by

28 $${\overline{Q}}_{*}({s}_{t},{a}_{t},{\beta }_{t+1}): 	\!\!=\mathop{\sum}\limits_{{s}_{t+1}^{\prime}}{P}_{{a}_{t}}({s}_{t},{s}_{t+1}^{\prime})({R}_{{a}_{t}}({s}_{t},{s}_{t+1}^{\prime})+\gamma {\overline{V}}_{*}({s}_{t+1}^{\prime},{\beta }_{t+1}))\\ 	=\mathop{\sum}\limits_{{s}_{t+1}^{\prime}}{P}_{{a}_{t}}({s}_{t},{s}_{t+1}^{\prime}){R}_{{a}_{t}}({s}_{t},{s}_{t+1}^{\prime})+\gamma \mathop{\sum}\limits_{{s}_{t+1}^{\prime}}{P}_{{a}_{t}}({s}_{t},{s}_{t+1}^{\prime}){\overline{V}}_{*}({s}_{t+1}^{\prime},{\beta }_{t+1}).$$

However, the direct calculation of the above expressions (essential in the forthcoming Learning process subsection) are non-feasible in the existing form, due to the enormous set of states ${s}_{t+1}^{\prime}$ that can be reached from state *s*_*t*_, even for relatively small networks (see the definition of the MDP for details on reachable states). In order to overcome this hurdle, we propose the following technique, which exploits the duality between the dynamics of the financial network (our nodes’ default modelling) and the MDP transition probabilities and rewards. To that end, we treat the two sums on the right-hand side of eq. (28) separately, and obtain the desired reformulation in the following three steps.

Step 1. We first recall (cf. the definition (21)) that ${\sum }_{s^{\prime} }{P}_{a}(s,{s}^{\prime}){R}_{a}(s,{s}^{\prime})$ is the one-step expected reward after taking action *a*∈*A*_*s*_. We then recall that, the transition probability ${P}_{a}(s,{s}^{\prime})$ was defined through the Gaussian latent variable model (see (17)) and observe that there is a one-to-one correspondence between additional nodes defaulting from state *s* and the resulting state ${s}^{\prime}$ that is reached given action *a*. In light of this duality, we can rewrite the first sum in terms of the nodes of the network instead of the MDP transition probabilities and rewards, according to

29 $$\mathop{\sum}\limits_{{s}_{t+1}^{\prime}}{P}_{a}({s}_{t},{s}_{t+1}^{\prime}){R}_{a}({s}_{t},{s}_{t+1}^{\prime})=-\mathop{\sum}\limits_{i\in {{{{{{{\mathcal{I}}}}}}}}\setminus {{{{{{{{\mathcal{I}}}}}}}}}_{def}(t)}P{D}_{i}^{a}(t){L}_{i}^{a}(t),$$

with the updated probability of default $P{D}_{i}^{a}(t)$ and taxpayers’ overall loss ${L}_{i}^{a}(t)$ after taking action ${a}_{t}\in {A}_{{s}_{t}}$ at time *t*, given by (13) and (16), respectively.

Step 2. The term ${\sum }_{s^{\prime} }{P}_{a}(s,{s}^{\prime})\ {\overline{V}}_{*}(s^{\prime},\beta )$ can then be estimated via Monte Carlo simulations, which involve (a) sampling ${s}^{\prime}$ using the distribution ${P}_{s}^{a}$ defined by the transition probability mass function ${P}_{a}(s,{s}^{\prime})$ and (b) calculating the expected value ${E}^{{P}_{s}^{a}}[{\overline{V}}_{*}(s^{\prime},\beta )]$ by averaging the values ${\overline{V}}_{*}(s^{\prime},\beta )$. Hence, we have

30 $$\mathop{\sum}\limits_{{s}_{t+1}^{\prime}}{P}_{{a}_{t}}({s}_{t},{s}_{t+1}^{\prime}){\overline{V}}_{*}({s}_{t+1}^{\prime},{\beta }_{t+1}) \sim {E}^{{P}_{{s}_{t}}^{{a}_{t}}}[{\overline{V}}_{*}({s}_{t+1}^{\prime},\beta )].$$

However, the non-feasibility is essentially still present here due to the enormous number of states ${s}_{t+1}^{\prime}$ involved in ${P}_{{a}_{t}}({s}_{t},{s}_{t+1}^{\prime})$, which defines the sampling distribution ${P}_{{s}_{t}}^{{a}_{t}}$.

Similarly to Step 1, we once again use our knowledge of the underlying network dynamics to describe the right hand side of (30) in terms of nodes defaulting instead of MDP transition probabilities. Using the duality between states ${s}_{t+1}^{\prime}$ reached given action *a*_*t*_ and the set ${{{{{{{{\mathcal{I}}}}}}}}}_{def}(t+1)\setminus {{{{{{{{\mathcal{I}}}}}}}}}_{def}(t)$ of additional nodes defaulting at time *t* + 1, we denote by ${Q}_{{s}_{t}}^{{a}_{t}}$ the probability distribution of states ${s}_{t+1}^{\prime}$, which are derived by using our Gaussian latent variable model. In particular, sampling from distribution ${Q}_{{s}_{t}}^{{a}_{t}}$ translates into first simulating which nodes *i* default at time *t* + 1, i.e. $i\in {{{{{{{{\mathcal{I}}}}}}}}}_{def}(t+1)\setminus {{{{{{{{\mathcal{I}}}}}}}}}_{def}(t)$, via the updated default mechanism in (14), and then we obtain the corresponding state ${s}_{t+1}^{\prime}$ with all its resulting characteristics. Given that the distributions ${Q}_{s}^{a}$ and ${P}_{s}^{a}$ are equivalent (due to the aforementioned duality/one-to-one correspondence), we can therefore rewrite (30) as

31 $$\mathop{\sum}\limits_{{s}_{t+1}^{\prime}}{P}_{{a}_{t}}({s}_{t},{s}_{t+1}^{\prime}){\overline{V}}_{*}({s}_{t+1}^{\prime},{\beta }_{t+1}) \sim {E}^{{Q}_{{s}_{t}}^{{a}_{t}}}[{\overline{V}}_{*}({s}_{t+1}^{\prime},\beta )].$$

Step 3. Merging the expressions obtained in Steps 1 and 2, namely (29) and (31), we can eventually rewrite ${\overline{Q}}_{*}({s}_{t},{a}_{t},{\beta }_{t+1})$ from (28) for all *t* ∈ [0, *M*−1], in the form of

32 $${\overline{Q}}_{*}({s}_{t},{a}_{t},{\beta }_{t+1})=-\mathop{\sum}\limits_{i\in {{{{{{{\mathcal{I}}}}}}}}\setminus {{{{{{{{\mathcal{I}}}}}}}}}_{def}(t)}P{D}_{i}^{{a}_{t}}{L}_{i}^{{a}_{t}}+\gamma {E}^{{Q}_{{s}_{t}}^{{a}_{t}}}\left[{\overline{V}}_{*}({s}_{t+1}^{\prime},{\beta }_{t+1})\right].$$

### Learning process

In order to learn the parameters *β*_*ik*_(*t*) we primarily need to use the Bellman optimality equation from (26). Recalling the expression (25) leading to its original derivation and using the approximation ${\overline{Q}}_{*}({s}_{t},{a}_{t},{\beta }_{t+1})$ from (28) instead of Q_*_(*s*_*t*_, *a*_*t*_), we can define a function *V*_*B*_, that we call Bellman value, by

$${V}_{B}({s}_{t},{\beta }_{t+1}): 	=\mathop{\max }\limits_{{a}_{t}}\left\{{\overline{Q}}_{*}({s}_{t},{a}_{t},{\beta }_{t+1})\right\}\\ 	=\mathop{\max }\limits_{{a}_{t}}\left\{\mathop{\sum}\limits_{{s}_{t+1}^{\prime}}{P}_{{a}_{t}}({s}_{t},{s}_{t+1}^{\prime})\left({R}_{{a}_{t}}({s}_{t},{s}_{t+1}^{\prime})+\gamma {\overline{V}}_{*}({s}_{t+1}^{\prime},{\beta }_{t+1})\right)\right\}.$$

or equivalently, using (32), we get the more computationally convenient (recall our proposed technique in the Action value function approximation subsection) form

33 $${V}_{B}({s}_{t},{\beta }_{t+1}): \!\!=\mathop{\max }\limits_{{a}_{t}}\left\{-\mathop{\sum}\limits_{i\in {{{{{{{\mathcal{I}}}}}}}}\setminus {{{{{{{{\mathcal{I}}}}}}}}}_{def}(t)}P{D}_{i}^{{a}_{t}}{L}_{i}^{{a}_{t}}+\gamma {E}^{{Q}_{{s}_{t}}^{{a}_{t}}}\left[{\overline{V}}_{*}({s}_{t+1}^{\prime},{\beta }_{t+1})\right]\right\}.$$

Our learning process will then compare our approximation ${\overline{V}}_{*}({s}_{t},{\beta }_{t})$ from (27) of the optimal value function with *V*_*B*_(*s*_*t*_, *β*_*t*+1_) from (33), at each state *s*_*t*_ and at any time *t*, with the aim of adjusting *β* so that the two values come closer. A potential issue here is that the Bellman value *V*_*B*_ depends itself on *β*, which is the parameter we want to fit, hence potentially triggering a divergent loop. In order to guarantee the convergence of our approach, we thus need to use specific learning strategies.

To begin the procedure, we can initialise *β* with *β*_*ik*_(*t*) = 1 for all *i*, *k*, as a natural starting point due to the intuition behind our initial approximation ${\overline{V}}_{*}({s}_{t},{\beta }_{t})$ of the optimal value function in (27), where *β*_*ik*_(*t*) multiply the approximated expected direct losses ${\overline{Z}}_{ik}({s}_{t})$ (recall the Value function approximation and parametrisation subsections for more details). Then we notice that, for each state *s*_*t*_, our approximation ${\overline{V}}_{*}({s}_{t},{\beta }_{t})$ depends on *β* at time *t*, while the corresponding *V*_*B*_(*s*_*t*_, *β*_*t*+1_) on *β* at time *t* + 1. Using this fact, in order to guarantee the convergence of our learning process, we fit *β* backwards in time. This results in ${\overline{V}}_{*}({s}_{t},{\beta }_{t})$ being compared at time *t* with a value *V*_*B*_(*s*_*t*_, *β*_*t*+1_) that is fixed, because *β*_*t*+1_ has already been fitted in the previous step (time *t* + 1), thus solving any potential convergence problem. This learning process then repeats the same procedure backwards in time by performing a ridge regression comparing ${\overline{V}}_{*}$ with *V*_*B*_, by fitting *β* until the difference between them is “small enough”, and the procedure then concludes successfully with obtaining *β*^*fit*^, i.e. the fitted parameters *β*_*t*_ for each time *t*.

It is worth noting that our aforementioned approach is feasible due to the facts that: (a) the crisis has a fixed maturity *M*, and (b) the value function V_*_(*s*_*M*_) = 0 at time *M* by definition (19). Consequently, we observe that V_*_(*s*_*t*_) = 0 for all *t* ≥ *M*, while for *t* ∈ [0, *M*−1], the backwards procedure works as follows.

The fact that V_*_(*s*_*M*_) = 0 further implies that at time *M*−1, we have *V*_*B*_(*s*_*M*−1_, *β*_*M*_) ≡ *V*_*B*_(*s*_*M*−1_), since it will not depend on *β*. In view of (33), we can thus write

34 $${V}_{B}({s}_{M-1})=\mathop{\max }\limits_{{a}_{M-1}}\left\{-\mathop{\sum}\limits_{i\in {{{{{{{\mathcal{I}}}}}}}}\setminus {{{{{{{{\mathcal{I}}}}}}}}}_{def}(M-1)}P{D}_{i}^{{a}_{M-1}}{L}_{i}^{{a}_{M-1}}\right\}={V}_{*}({s}_{M-1}),$$

where the latter equality follows from (26) and (29).

Now that we can calculate the exact optimal value function V_*_ for each state at time *M*−1, we notice from (33) that *V*_*B*_(*s*_*M*−2_, *β*_*M*−1_) ≡ *V*_*B*_(*s*_*M*−2_) is also independent of *β*, namely

35 $${V}_{B}({s}_{M-2})=\mathop{\max }\limits_{{a}_{M-2}}\left\{-\mathop{\sum}\limits_{i\in {{{{{{{\mathcal{I}}}}}}}}\setminus {{{{{{{{\mathcal{I}}}}}}}}}_{def}(M-2)}P{D}_{i}^{{a}_{M-2}}{L}_{i}^{{a}_{M-2}}+\gamma {E}^{{Q}_{{s}_{M-2}}^{{a}_{M-2}}}\left[{V}_{*}({s}_{M-1}^{\prime})\right]\right\}.$$

We then fit *β* backwards in time for the decreasing sequence of time steps (*M*−2,…,0), by creating a representative portfolio *S*_*R*_ of MDP states for each time step (namely, a subset of the state space *S* that is reachable from *s*_0_, see the following two subsections for details) and performing a ridge regression (with a 5-fold cross-validation) comparing ${\overline{V}}_{*}({s}_{t},{\beta }_{t})$ with *V*_*B*_(*s*_*t*_, *β*_*t*+1_). To be more precise, for time step *M*−2, we compare ${\overline{V}}_{*}({s}_{M-2},{\beta }_{M-2})$ with *V*_*B*_(*s*_*M*−2_), for all the states *s*_*M*−2_∈*S*_*R*_, and we fit *β*_*M*−2_.

Then, for time step *M*−3, we calculate from (33) that

36 $${V}_{B}({s}_{M-3},{\beta }_{M-2})=\mathop{\max }\limits_{{a}_{M-3}}\left\{-\mathop{\sum}\limits_{i\in {{{{{{{\mathcal{I}}}}}}}}\setminus {{{{{{{{\mathcal{I}}}}}}}}}_{def}(M-3)}P{D}_{i}^{{a}_{M-3}}{L}_{i}^{{a}_{M-3}}+\gamma {E}^{{Q}_{{s}_{M-3}}^{{a}_{M-3}}}\left[{\overline{V}}_{*}({s}_{M-2}^{\prime},{\beta }_{M-2})\right]\right\},$$

then compare it with ${\overline{V}}_{*}({s}_{M-3},{\beta }_{M-3})$ and hence fit *β*_*M*−3_, for all the states *s*_*M*−3_∈*S*_*R*_.

We continue the procedure backwards in time until we successfully obtain *β*^*fit*^, i.e. the fitted *β*_*t*_ for each time *t*.

### “Reachable” MDP states example

To illustrate the implementation of our model and how to identify reachable states, we consider here a simple example of a network with three nodes ${{{{{{{\mathcal{I}}}}}}}}=\{1,2,3\}$ and *w*_*ij*_ = 1, for all $i\; \ne \,j\in {{{{{{{\mathcal{I}}}}}}}}$, at a time *t*. To define state *s*_*t*_ at time t, we assume that node $3\in {{{{{{{{\mathcal{I}}}}}}}}}_{def}(t)$ has already defaulted, while the remaining nodes have *W*_*i*_(*t*) = 100, *E*_*i*_(*t*) = 3 and *PD*_*i*_(*t*) = 0.001 for $i\in {{{{{{{\mathcal{I}}}}}}}}\setminus {{{{{{{{\mathcal{I}}}}}}}}}_{def}(t)=\{1,2\}$.

In case the government does not intervene, the states ${s}_{t+1}^{\prime}$ that can be reached from state *s*_*t*_ are those where: (i) all the nodes default at time *t*, i.e. ${{{{{{{{\mathcal{I}}}}}}}}}_{def}(t+1)={{{{{{{\mathcal{I}}}}}}}}$; (ii) nodes 1 and 2 are still active and *W*_*i*_(*t* + 1), *E*_*i*_(*t* + 1) and *PD*_*i*_(*t* + 1) for *i* ∈ {1, 2} are the same as for state *s*_*t*_; (iii) node 1 defaults at time *t* while node 2 remains active, i.e. ${{{{{{{{\mathcal{I}}}}}}}}}_{def}(t+1)=\{1,3\}$, *W*_2_(*t* + 1) = 99 and *E*_2_(*t* + 1) = 2 (since the impact *I*_2_(*t*) = *w*_21_ = 1) and *PD*_2_(*t* + 1) needs to take the value calculated via (6) using the *W*_2_(*t* + 1) and *E*_2_(*t* + 1) inputs; and (iv) node 2 defaults at time *t* but node 1 remains active, which is analogous to (iii) by swapping indices 1 and 2.

Now, if the government decides to invest, i.e. $a\to (\Delta {J}_{1}^{a}(t),\Delta {J}_{2}^{a}(t))$ on nodes 1 and 2, respectively, at time *t*, we need to update the capitals ${E}_{i}^{a}(t)={E}_{i}(t)+\Delta {J}_{i}^{a}(t)$ and total assets ${W}_{i}^{a}(t)={W}_{i}(t)+\Delta {J}_{i}^{a}(t)$ for *i* ∈ {1, 2} according to the government intervention (cf. (12)) and then use ${E}_{i}^{a}(t),{W}_{i}^{a}(t)$ to calculate the updated $P{D}_{i}^{a}(t)$ according to (13). Using these updated characteristics ${E}_{i}^{a}(t)$,${W}_{i}^{a}(t)$ and $P{D}_{i}^{a}(t)$, we can then perform the same analysis as above to identify the reachable states.

### Representative portfolio *S*_*R*_ of “reachable” states

Recall that in the Value function approximation subsection, we express our approximation ${\overline{V}}_{*}({s}_{t},\beta )$ in (27) of the optimal value function V_*_(*s*_*t*_) as a linear combination of terms ${\overline{Z}}_{ik}({s}_{t})$ with coefficients *β*_*ik*_(*t*). In order to fit these *β*_*ik*_(*t*), our methodology in the Learning process subsection, requires the identification of a representative portfolio *S*_*R*_ of MDP states that can be reached at time *t* from the initial state *s*_0_, and for which we can calculate the Bellman value *V*_*B*_ using (33), equate it with ${\overline{V}}_{*}({s}_{t},{\beta }_{t})$ and derive a set of linear equations in order to obtain the coefficients *β*_*ik*_(*t*) via a ridge regression.

The states *s*_*t*_ in our representative portfolio *S*_*R*_ at each time t, are obtained in two main ways, taking into account the trade-off between stable results and computational resources.

1^*st*^ way. We obtain elements *s*_*t*_ ∈ *S*_*R*_ from the initial state *s*_0_, after changing the time to maturity from *M* to *M*−*t* (i.e. state *s*_0_ is moved forward in time) and forcing a set *U* of nodes to default. The representative portfolio *S*_*R*_ contains:

- the state corresponding to $U={{\emptyset}}$;
- all the states corresponding to *U* = {*i*} for $i\in {{{{{{{\mathcal{I}}}}}}}}$, i.e. with one additional defaulted node with respect to *s*_0_;
- a selection of states corresponding to ∣*U*∣ > 1, i.e. with multiple additional defaulted nodes—these are chosen randomly with probabilities proportional to *exp*(−∣*U*∣), so that a greater importance is given to states with fewer number of additional defaults, as they are more likely to be reached in an actual simulation.

2^*nd*^ way. In addition to the above states, we obtain elements *s*_*t*_∈*S*_*R*_ by performing a government action ${a}_{0}\in {A}_{{s}_{0}}$ on state *s*_0_ and then move the corresponding state forward at time *t*, i.e. make the time to maturity equal to *M*−*t*.

### Optimal solution of the MDP

Finally, we use the (fitted) optimal value function ${\overline{V}}_{*}(s,{\beta }^{fit})$, with *β*^*fit*^ obtained in the Learning process subsection, together with (32) to calculate ${\overline{Q}}_{*}(s,a,{\beta }^{fit})$, hence solving the MDP. The resulting optimal action value function is

37 $${Q}_{*}({s}_{t},{a}_{t}) \,\approx \, {\overline{Q}}_{*}({s}_{t},{a}_{t},{\beta }^{fit})=-\mathop{\sum}\limits_{i\in {{{{{{{\mathcal{I}}}}}}}}\setminus {{{{{{{{\mathcal{I}}}}}}}}}_{def}(t)}P{D}_{i}^{{a}_{t}}{L}_{i}^{{a}_{t}}+\gamma {E}^{{Q}_{{s}_{t}}^{{a}_{t}}}\left[{\overline{V}}_{*}({s}_{t+1}^{\prime},{\beta }^{\,fit})\right].$$

## Data Availability

The data used in this study are available in public databases whose web links are provided in citations.

## Code availability

Our research concerns a theoretical framework to solve a complex MDP that is relevant for financial systemic risk analysis. The readers can access a full mathematical description of our artificial intelligence technique and all its mathematical technicalities and their resolutions to validate our approach in the Methods section.
